# Inland water greenhouse gas emissions offset the terrestrial carbon sink in the northern cryosphere

**DOI:** 10.1126/sciadv.adp0024

**Published:** 2024-09-27

**Authors:** Chunlin Song, Shaoda Liu, Genxu Wang, Liwei Zhang, Judith A. Rosentreter, Gang Zhao, Xiangyang Sun, Yuanzhi Yao, Cuicui Mu, Shouqin Sun, Zhaoyong Hu, Shan Lin, Juying Sun, Yang Li, Ying Wang, Yuhao Li, Peter A. Raymond, Jan Karlsson

**Affiliations:** ^1^ State Key Laboratory of Hydraulics and Mountain River Engineering, College of Water Resource and Hydropower, Sichuan University, Chengdu, Sichuan 610065, China.; ^2^ State Key Laboratory of Water Environment Simulation, School of Environment, Beijing Normal University, Beijing 100875, China.; ^3^ State Key Laboratory of Estuarine and Coastal Research, Yangtze Delta Estuarine Wetland Ecosystem Observation and Research Station, East China Normal University, Shanghai, China.; ^4^ Center for Coastal Biogeochemistry, Faculty of Science and Engineering, Southern Cross University, Lismore, New South Wales, Australia.; ^5^ Institute of Geographic Sciences and Natural Resources Research, Chinese Academy of Sciences, Beijing 100101, China.; ^6^ School of Geographic Sciences, East China Normal University, Shanghai, China.; ^7^ Key Laboratory of Western China’s Environmental Systems (Ministry of Education), College of Earth and Environmental Sciences, Observation and Research Station on Eco-Environment of Frozen Ground in the Qilian Mountains, Lanzhou University, Lanzhou, China.; ^8^ School of the Environment, Yale University, New Haven, CT 06511, USA.; ^9^ Climate Impacts Research Centre (CIRC), Department of Ecology and Environmental Science, Umeå University, Linnaeus väg 6, 901 87 Umeå, Sweden.

## Abstract

Climate-sensitive northern cryosphere inland waters emit greenhouse gases (GHGs) into the atmosphere, yet their total emissions remain poorly constrained. We present a data-driven synthesis of GHG emissions from northern cryosphere inland waters considering water body types, cryosphere zones, and seasonality. We find that annual GHG emissions are dominated by carbon dioxide ($1149.2_{1004.8}^{1307.5}$ teragrams of CO_2_; ${\text{median}}_{Q1}^{Q3}$) and methane ($14.2_{10.1}^{18.5}$ teragrams of CH_4_), while the nitrous oxide emission ($5.4_{-1.4}^{12.2}$ gigagrams of N_2_O) is minor. The annual CO_2_–equivalent (CO_2_e) GHG emissions from northern cryosphere inland waters total $1.5_{1.3}^{1.8}$ or $2.3_{1.8}^{2.8}$ petagrams of CO_2_e using the 100- or 20-year global warming potentials, respectively. Rivers emit 64% more CO_2_e GHGs than lakes, despite having only one-fifth of their surface area. The continuous permafrost zone contributed half of the inland water GHG emissions. Annual CO_2_e emissions from northern cryosphere inland waters exceed the region’s terrestrial net ecosystem exchange, highlighting the important role of inland waters in the cryospheric land-aquatic continuum under a warming climate.

## Teaser

Lakes and rivers in the northern cryosphere may emit more greenhouse gases than the terrestrial carbon dioxide uptake.

## INTRODUCTION

The northern hemisphere permafrost (*1*) and glacier (*2*) distribution areas, which are integral parts of the northern cryosphere, have undergone notable degradation due to global warming. Specifically, the northern cryosphere warms two to four times faster than the global average (*3*–*6*), profoundly affecting the biogeochemical functions of inland water ecosystems (*7*). Covering one-fourth of the northern hemisphere land (*8*), the northern permafrost soil contains 1014 Pg of organic carbon and 67 Pg of nitrogen to a depth of 3 m (*9*, *10*), which is nearly half of the global soil organic carbon and nitrogen stock (*9*, *11*). However, the large carbon and nitrogen stocks in frozen permafrost are vulnerable to thawing. When permafrost thaws and glaciers melt, large quantities of mobilized organic matter can be decomposed, which can lead to substantial release of carbon dioxide (CO_2_), methane (CH_4_), and nitrous oxide (N_2_O) to the atmosphere (*11*, *12*). A substantial portion of the mobilized organic matter and greenhouse gases (GHGs) from permafrost thawing is exported to surrounding inland waters, causing additional emissions of GHGs via enhanced decomposition and outgassing processes (*13*–*18*). The northern cryosphere is also a waterscape-abundant region (*19*) accounting for nearly one-third (table S1) of the surface area of global lakes, ponds, reservoirs, rivers, and streams (collectively as inland waters) (*20*–*22*), therefore play an important role in global land-aquatic C and N cycles. Since the discovery of CO_2_ supersaturation in Arctic lakes and streams (*23*, *24*), numerous studies from the Arctic, boreal, and Qinghai–Tibet Plateau (QTP) regions have shown that cryosphere inland waters are important atmospheric GHG sources (*13*, *14*, *25*–*30*). However, the magnitude of total GHG emissions from northern cryospheric inland water bodies remains highly uncertain, presumably as a result of major gaps in data sources for flux rates and inconsistent water surface areas, upscaling methods, and geographical extents used (*13*, *31*–*33*). Although rivers and lakes in the cryosphere may disproportionately contribute to global inland water’s GHG emission (*22*, *34*–*37*), regionalized estimates of their combined GHG (CO_2_ + CH_4_ + N_2_O) emissions have so far been lacking. Furthermore, from a land-aquatic continuum perspective (*38*), the aquatic GHG emissions may offset a considerable portion of the terrestrial carbon sink (*39*–*41*). Current Earth System Models lack to adequately integrate inland water GHG processes in cryospheric regions, meaning that these regions are likely underrepresented in modeling efforts (*42*). As such, a holistic and accurate quantification of the emissions of the three major GHGs from northern cryosphere inland waters is needed to facilitate current and future GHG radiative assessments and to constrain net watershed carbon and nitrogen balances.

**Fig. 1.**
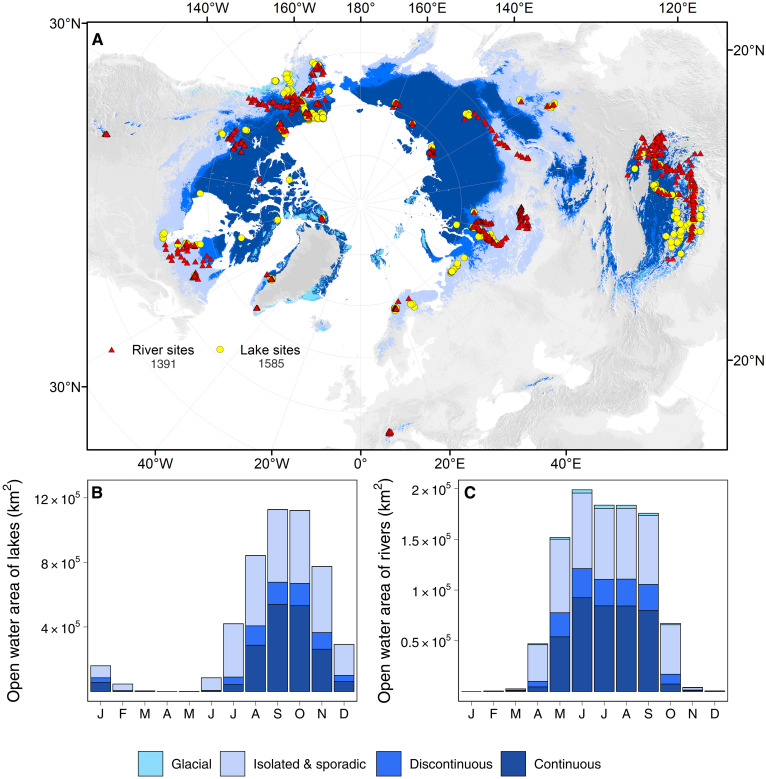
Inland water GHG study sites and open water surface area of the northern cryosphere. (**A**) Spatial distribution of GHG observation sites of lakes and rivers across the northern land cryosphere region. The northern hemisphere permafrost and glacier base maps are from refs. (*1*) and (*2*). (**B** and **C**) Monthly open water surface area of lakes and rivers of the cryosphere (see Materials and Methods).

**Fig. 2.**
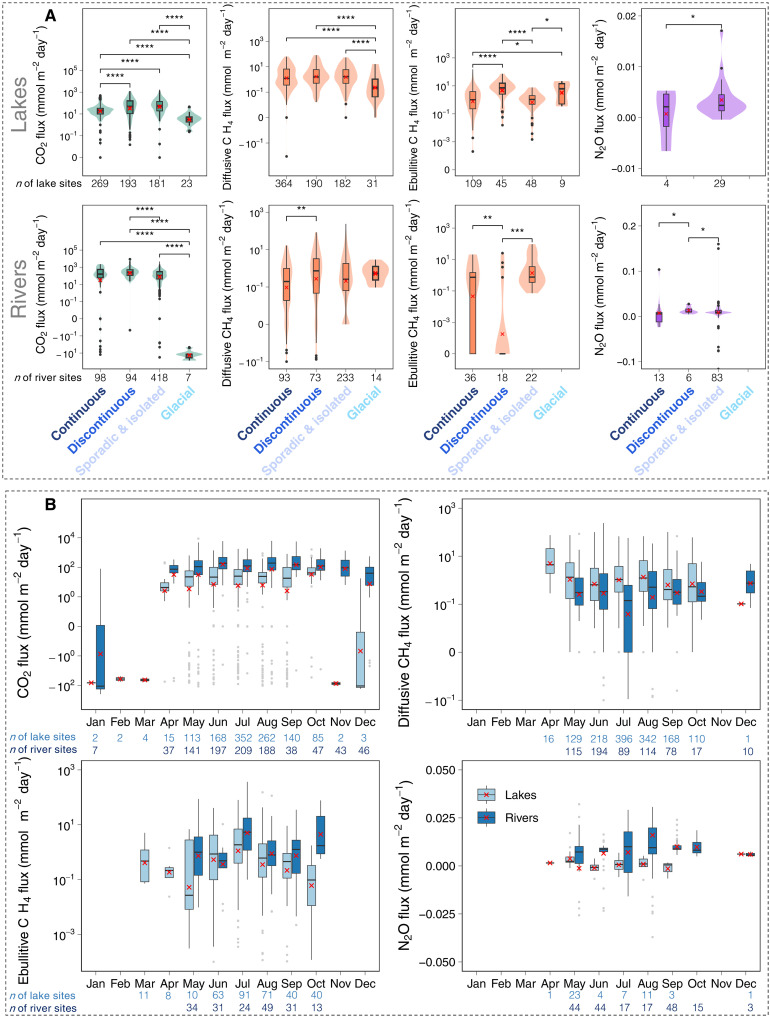
Northern cryosphere inland water GHG flux rates. (**A**) Box and violin plots showing upper and lower quartiles and median areal GHG flux rates (mmol m^−2^ day^−1^) for lakes (upper four panels) and rivers (lower four panels) of different cryosphere zones. The red crosses indicate the mean values. The asterisks between the paired groups indicate the statistical significance of Wilcoxon test results: *P ≤ 0.05; **P ≤ 0.01; ***P ≤ 0.001; ****P ≤ 0.0001. (**B**) Seasonal variability of inland water GHG flux rates. Boxplots show upper quartile, lower quartile, and median monthly diffusive CO_2_, CH_4_, and N_2_O and ebullitive CH_4_ areal flux rates for lakes and rivers. The red crosses indicate the mean values. Curves are fitted with locally estimated scatterplot smoothing curves to show the seasonal variations in GHG emissions for lakes and rivers. Note that the *y* axes for CO_2_ and CH_4_ fluxes are log-transformed. The number of sites for each cryosphere zone or month is indicated below the figures.

Here, we present a data-driven synthesis of CO_2_, CH_4_, and N_2_O emissions from inland waters in the northern cryosphere region, defined as the permafrost (*1*) and glacier (*2*) landscapes in the northern hemisphere. We categorized the northern cryosphere into four cryosphere zones using the boundaries defined in permafrost and glacier extent maps (*1*, *2*), i.e., (i) glacial region, (ii) sporadic and isolated permafrost, (iii) discontinuous permafrost, and (iv) continuous permafrost. We compiled a large dataset that incorporates spatiotemporal-resolved measurements of CO_2_, CH_4_, and N_2_O concentrations and fluxes in cryosphere inland waters from the literature published until April 2023 (*43*). The dataset encompasses 1585 sites of lakes, ponds, and reservoirs (hereafter referred to as “lakes”) and 1391 sites of rivers and streams (hereafter referred to as “rivers”) with GHGs measured between the years 1983 and 2022 (figs. S1 to S3), providing extensive spatial coverage (Fig. 1A) and the whole open water period data, which captured the GHGs’ seasonality in this region (Fig. 2 and fig S4). This study considers different water body types (lakes and rivers), seasonality, and all three major GHGs compared to previous studies that were specific to the Arctic (*13*, *15*) or QTP region (*44*) and also did not include seasonality and/or inland water types. We revised these previous estimates by leveraging monthly dynamic areal coverage of northern cryospheric lakes and rivers (*22*, *45*) along with more GHG measurements to reduce the uncertainties induced by the seasonality of inland water surface area and data scarcity. Our data compilation allows us to differentiate GHG emissions spatially from four cryosphere zones, which is critical for regional GHG budgets. To compare the radiative forcing of the three gases, we calculated CO_2_-equivalent (CO_2_e) CH_4_ and N_2_O emissions using the 20- and 100-year global warming potentials (GWP_20_ and GWP_100_). Last, we compared our cryosphere CO_2_e GHG inland water emissions with the terrestrial-atmospheric net ecosystem CO_2_ exchange (NEE) of this region to estimate the relative importance of inland waters in the cryosphere carbon cycle.

## RESULTS AND DISCUSSION

### Variability of inland water area and GHG fluxes

Expectedly, northern cryosphere inland waters exhibit strong seasonal variations in hydrology and ice phenology, which strongly affects their seasonal open water surface area available for GHG outgassing (Fig. 1). Therefore, it is imperative to consider both seasonal and spatial variability of the GHG flux rate and the open surface area when estimating total annual GHG emissions from the cryosphere. To facilitate monthly upscaling, we curated the monthly open water surface area of cryosphere rivers and lakes from two recent studies (*22*, *45*). For lakes, we also extrapolated the area of small lakes from 0.1 km^2^ to a minimum size of 0.0001 km^2^ to achieve a more complete account of the GHG emission from small lakes (see Materials and Methods). The open water surface area exhibits distinct changes over the months for both lakes (Fig. 1B) and rivers (Fig. 1C) (table S1). Lake open water surface area reaches its peak between September and October and drops to a minimum between March and May. River open water surface area rises in May, peaks in June, and gradually declines until winter. These seasonal changes of riverine open water surface area reflect the combined effects of snowmelt, precipitation, and ice phenology. Compared to rivers, the lag observed in lake open water surface area indicates the distinct thermal regime of lakes and their differing ice phenology behaviors.

Inland water GHG flux rates vary greatly across different cryosphere regions (Fig. 2A and table S2). Permafrost lakes and rivers exhibit over 10 times higher CO_2_ flux rates than glacial lakes and rivers (Fig. 2A and table S2). This discrepancy is likely to be attributed to the substantial carbon stocks in permafrost soils and peatlands that are partly mobilized, processed, and released from recipient inland waters. A comparison between permafrost zones shows that CO_2_ flux rates in continuous permafrost regions are lower than those of discontinuous permafrost regions. This is likely caused by the lower temperatures and therefore lower organic C mineralization rates and less terrestrial C export in the continuous permafrost zone compared to discontinuous permafrost zones (*28*, *46*). Notably, diffusive and ebullitive CH_4_ flux rates are markedly different across permafrost regions (Fig. 2). The highest river ebullitive CH_4_ flux was found in sporadic/isolated permafrost, mostly because of the high ebullition rates found in the high-elevation and shallow QTP rivers characterized by abundant organic matter, cold-tolerant methanogens, and low air pressure systems that create favorable conditions for CH_4_ ebullition (*25*). The highest lake ebullitive CH_4_ flux occurs in discontinuous permafrost, which is consistent with a previous study (*13*). Other factors, including soil carbon richness, water depth, topography, and vegetation, may also affect the spatial patterns of inland water CO_2_ and CH_4_ flux rates (*27*, *44*). It is worth noting that, in both lakes and rivers, zero ebullition measurements were reported more often than non-zero ebullitive CH_4_ fluxes (fig. S5), highlighting the episodic nature of CH_4_ ebullition. Although CH_4_ ebullition dominates total CH_4_ emissions in most lake and river systems (*25*, *33*), unexpectedly, 39% of river measurements and 13% of lake measurements in our dataset reports zero CH_4_ ebullition. This emphasizes the need for further studies to improve our mechanistic understanding of CH_4_ ebullition in these cold regions. Both lakes (65%) and rivers (36%) exhibit undersaturation in N_2_O (figs. S5 and S6), suggesting that cryosphere inland waters act predominantly as N_2_O sinks as was found in boreal aquatic networks in Canada (*29*).

Considering all seasonally available data, we find that CO_2_, CH_4_, and N_2_O fluxes exhibit pronounced but nonuniversal seasonal variation patterns (Fig. 2B). Using subsets of the river and lake sites that have at least 5 months of observations, we also find distinct but inconsistent seasonal patterns in GHGs from lakes and rivers (figs. S7 and S8), suggesting the complex regulatory processes behind the seasonality of GHGs. For example, the high diffusive CH_4_ fluxes from lakes during April and May (Fig. 2B and fig. S7) are likely to be an “ice-out effect,” which describes the burst of winter accumulated gas during the short ice-thaw period (*27*, *47*–*49*). However, this phenomenon is not obvious in rivers and for other gas types. At study locations where both CH_4_ diffusion and ebullition are available, we find that lakes and rivers have contrasting ebullitive contributions through seasons (fig. S9). While the relative contribution of ebullition to total CH_4_ fluxes in lakes peaks in June and gradually decreases until October, the contribution of ebullition in rivers reaches its peak during the shoulder seasons (May and October). The higher relative contribution of CH_4_ diffusion in rivers during June and July is likely related to the increased discharge and external carbon inputs. Seasonality is a key feature of cryosphere inland waters that encompass strong seasonal changes in temperature, precipitation, hydrology, ice phenology, and primary productivity. All these changes inevitably alter the biogeochemical and physical processes of GHG production, transport pathways, outgassing, and consumption.

### Rivers dominate cryospheric inland water CO_2_ emissions

**Fig. 3.**
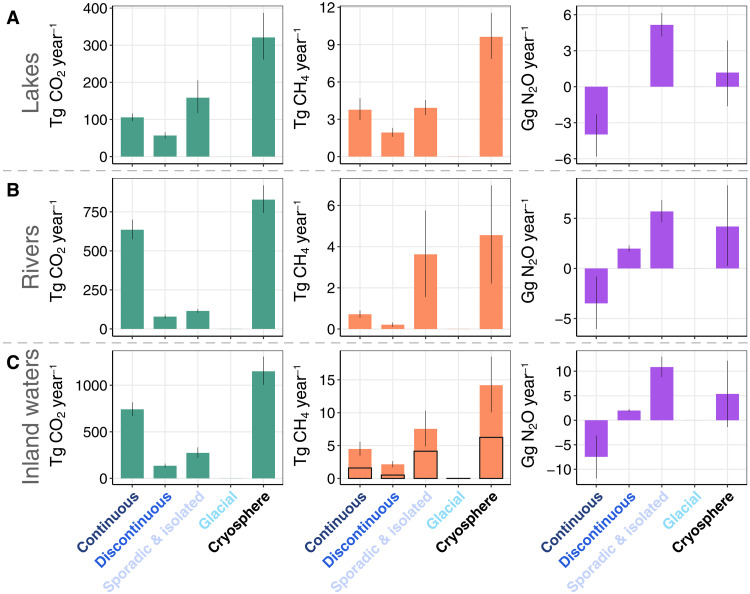
Annual GHG emissions of northern cryosphere inland waters segregated by cryosphere zones. Barplots showing the (median, Q1 to Q3) annual GHG emissions in northern cryosphere (**A**) lakes, (**B**) rivers, and (**C**) inland waters (i.e., lakes and rivers combined). The black pillars in the CH_4_ plots indicate the ebullition portions of the total CH_4_ emissions.

We estimate that the northern cryosphere lakes emit median (Q1 to Q3) 321 (261 to 387) Tg CO_2_ year^−1^ (Fig. 3A and Table 1). Our median lake emissions represent substantial 37% of global lake and reservoir CO_2_ emissions (*36*). This proportion is close to the area proportion of northern cryosphere lakes (table S1) compared to the total area of lakes worldwide. Although the ice-covered season in these lakes is longer than of lakes in other regions of the world, their comparatively higher areal CO_2_ flux rates (fig. S6) (*21*) lead to these high CO_2_ emissions. Compared to lakes in tropical regions where higher temperature enhances internal primary production and limits CO_2_ outgassing (*50*), our estimated annual CO_2_ emission from northern cryosphere lakes is five times higher than that in pan-tropical (24°S to 24°N) lakes (*50*). Among the different northern cryosphere zones, sporadic/isolated permafrost lakes contribute 49% of CO_2_ emissions, followed by continuous permafrost lakes (33%). The high CO_2_ emissions from sporadic/isolated permafrost lakes are likely caused by the large cumulative open water surface area throughout the year (table S1). Although continuous permafrost lakes have the largest surface area, their ice cover periods are longer than those of other regions. Lakes in glacial regions are generally small CO_2_ sources (Fig. 3A and Table 1) or even CO_2_ sinks as reported in recent studies (*51*, *52*).

**Table 1.** Annual GHG emissions from inland waters of different northern cryosphere zones. Numbers are given as medians (Q1 to Q3). NA, not available.

Water bodies	Cryosphere zones	CO_2_ Tg CO_2_ year^−1^	CH_4_ Tg CH_4_ year^−1^	CH_4_ Tg CO_2_e year^−1^ GWP20	CH_4_ Tg CO_2_e year^−1^ GWP100	N_2_O Gg N_2_O year^−1^	N_2_O Tg CO_2_e year^−1^ GWP20	N_2_O Tg CO_2_e year^−1^ GWP100	GHG Tg CO_2_e year^−1^ GWP20	GHG Tg CO_2_e year^−1^ GWP100
Lakes	Continuous	105.7 (95.8–116.3)	3.77 (2.92–4.69)	300.14 (232.91–373.55)	101.68 (78.9–126.55)	−3.97 (−5.82–−2.3)	−1.09 (−1.59–−0.63)	−1.09 (−1.59–−0.63)	691.44 (616.74–771.49)	653.69 (587.98–724.14)
	Discontinuous	56.7 (48.5–65.5)	1.93 (1.59–2.31)	154.12 (126.64–183.96)	52.21 (42.9–62.32)	NA	NA	NA	95.78 (74.18–118.92)	84.78 (68.88–102.26)
	Sporadic and isolated	158.6 (117–205.3)	3.92 (3.34–4.55)	312.25 (265.83–362.5)	105.78 (90.05–122.8)	5.15 (4.19–6.15)	1.41 (1.14–1.68)	1.41 (1.14–1.68)	405.5 (228.72–588.43)	214.37 (146.81–284.95)
	Glacial	0.02 (0.01–0.03)	0.01 (0.01–0.01)	0.62 (0.48–0.75)	0.21 (0.16–0.26)	NA	NA	NA	−0.09 (−0.3–0.11)	−0.43 (−0.56–−0.29)
	Cryosphere	321.0 (261.4–387.2)	9.63 (7.85–11.55)	767.12 (625.86–920.77)	259.88 (212.02–311.93)	1.18 (−1.63–3.85)	0.32 (−0.45–1.05)	0.32 (−0.45–1.05)	1192.62 (919.26–1478.95)	952.41 (803.02–1111.05)
Rivers	Continuous	635.3 (574.9–700.1)	0.72 (0.55–0.9)	57.09 (43.49–71.61)	19.34 (14.73–24.26)	−3.48 (−6.04–−0.82)	−0.95 (−1.65–−0.22)	−0.95 (−1.65–−0.22)	404.75 (327.12–489.22)	206.29 (173.11–242.22)
	Discontinuous	78.6 (65.7–93.1)	0.21 (0.1–0.32)	16.64 (8.02–25.19)	5.64 (2.72–8.53)	1.98 (1.69–2.29)	0.54 (0.46–0.63)	0.54 (0.46–0.63)	210.82 (175.14–249.46)	108.91 (91.4–127.82)
	Sporadic and isolated	114.9 (103.6–127.6)	3.63 (1.55–5.76)	289.05 (123.87–458.97)	97.92 (41.96–155.49)	5.69 (4.6–6.82)	1.55 (1.25–1.86)	1.55 (1.25–1.86)	472.26 (383.97–569.48)	265.79 (208.19–329.78)
	Glacial	−0.6 (−0.7–−0.5)	0.01 (0.01–0.01)	0.51 (0.4–0.61)	0.17 (0.14–0.21)	NA	NA	NA	0.64 (0.49–0.78)	0.21 (0.17–0.29)
	Cryosphere	828.2 (743.4–920.3)	4.56 (2.21–6.98)	363.28 (175.79–556.38)	123.07 (59.55–188.48)	4.18 (0.24–8.3)	1.14 (0.07–2.27)	1.14 (0.07–2.27)	1088.44 (886.81–1309.02)	581.2 (472.97–700.18)
Inland waters	Cryosphere	1149.2 (1004.8–1307.5)	14.19 (10.06–18.53)	1130.4 (801.65–1477.15)	382.95 (271.57–500.41)	5.36 (−1.39–12.15)	1.46 (−0.38–3.32)	1.46 (−0.38–3.32)	2281.0 (1806.1–2788.0)	1533.6 (1276.0–1811.2)

CO_2_ emissions from northern cryosphere rivers amount to 828 (743 to 920) Tg CO_2_ year^−1^ (Fig. 3B and Table 1), which is 2.6 (2.4 to 2.8) times greater than those in lakes. Despite the surface area of rivers being only one-fifth that of lakes (table S1), the CO_2_ flux rates from rivers are 763% higher than from lakes (Fig. 2B and table S2). This difference can be attributed to the fact that river networks being closely connected to terrestrial ecosystems, which facilitates high rates of outgassing (*22*, *53*), while lakes typically have lower hydrologic connectivity with land, weak processing of terrigenous carbon (*54*) and lower areal CO_2_ flux rates. Our estimated cryosphere river emissions are 11 to 18% of global riverine CO_2_ emissions (*21*, *22*, *36*)—a percentage that is lower than the area proportion of northern cryosphere rivers (25%; table S1) (*22*) to the total area of global rivers (*22*, *55*). This is anticipated due to the lower CO_2_ flux rates (Fig. 1, B and C, and table S2) of northern cryosphere compared to global rivers (*22*). The continuous permafrost region is the largest contributor (77%) to cryospheric riverine CO_2_ emissions, followed by sporadic/isolated permafrost rivers (14%). Rivers in northern glacial regions are small CO_2_ sinks, potentially reflecting that dissolved CO_2_ can be consumed by carbonate and silicate weathering in glacier rivers (*52*). The lack of terrigenous organic matter sources in glacial-dominated catchments also limits the production of CO_2_ in these rivers (*52*, *56*).

### Lakes dominate cryospheric inland water CH_4_ emissions

The estimated total CH_4_ emissions (diffusion plus ebullition) from cryosphere lakes amount to 9.6 (7.9 to 11.6) Tg CH_4_ year^−1^ (Table 1), which is 12% of global median lake and reservoir CH_4_ emissions (*36*). This finding is unexpected because the surface area of cryosphere lakes (1.1 × 10^6^ km^2^; table S1) is one-third of the global lake area [3.2 × 10^6^ km^2^ (*20*)] and lakes are often considered critical CH_4_ sources (*13*). The relatively low annual emissions may be attributed to the shorter emission period of these lakes due to ice cover in winter and low temperature–induced low CH_4_ production. For comparison, the pan-tropical lakes (24°S to 24°N) emit ~50% (5.1 Tg CH_4_ year^−1^) CH_4_ compared to cryospheric lakes while occupying less than one-fifth (209,394 km^2^) of their area, probably caused by the enhanced methanogenesis in warmer waters of the pan-tropical lakes (*50*). Sporadic/isolated permafrost lakes (40%) and continuous permafrost lakes (38%) clearly dominate total CH_4_ emissions from the cryosphere.

The magnitude of total CH_4_ emissions from rivers is 4.6 (2.2 to 7.0) Tg CH_4_ year^−1^ (Table 1), 52% less than emissions from cryosphere lakes and ~15 to 17% of recent global estimates for rivers and streams (*57*–*59*). Most riverine CH_4_ emissions (80%) come from sporadic/isolated permafrost rivers (Table 1), despite continuous permafrost rivers exhibiting a higher open water surface area (table S1). This can be attributed to the high flux rates in sporadic/isolated permafrost rivers (table S2). Continuous permafrost rivers (16%) are the second-largest contributors to cryosphere river CH_4_ emissions, followed by minor emissions from discontinuous permafrost and glacial rivers.

Collectively, cryosphere inland waters emit 14.2 (10.1 to 18.5) Tg CH_4_ year^−1^, with nearly half of the emissions coming from ebullition. Ebullition is the main CH_4_ emission pathway for sporadic/isolated permafrost inland waters, while diffusion dominates the CH_4_ emissions in continuous and discontinuous permafrost inland waters (Fig. 3C). This discrepancy is likely caused by high ebullitive CH_4_ flux per unit area in sporadic/isolated permafrost inland waters. Although estimates vary between studies, lakes consistently release more methane than rivers globally (*33*, *58*, *59*). In this study, we find the same pattern except for the region of sporadic/isolated permafrost where total CH_4_ emissions from rivers are comparable to those from lakes.

### Minor N_2_O emissions from lakes and rivers

We estimate that N_2_O emissions from northern cryosphere lakes amount to 1.18 (−1.63 to 3.85) Gg N_2_O year^−1^ (Fig. 3A and Table 1), which is less than 1% of recent global lake emissions (*36*). This low N_2_O emission cannot be solely explained by missing data in discontinuous permafrost and glacial lakes because these lakes account for only 12% of the total cryosphere lake area during the peak season (table S1). Instead, the widespread undersaturation of the N_2_O concentration and negative N_2_O flux rates (figs. S5 and S6) counteract a part of the N_2_O outgassing (*29*, *60*). For example, we find that sporadic/isolated permafrost lakes are N_2_O sources [5.15 (4.19 to 6.15) Gg N_2_O year^−1^], but continuous permafrost lakes offset 77% of these emission by acting as N_2_O sinks (Table 1).

Cryosphere rivers emit 4.18 (0.24 to 8.3) Gg N_2_O year^−1^ (Fig. 3B and Table 1), which is ~3% of global riverine N_2_O emission (*36*) but double that of the modeled boreal rivers emission of 2.19 Gg N_2_O year^−1^ for the year 2016 using a process-based model (*37*). Our larger estimate can be explained by the increased number of N_2_O observations in cryospheric rivers during the recent decades (fig. S3). In addition, Yao *et al.* (*37*) suggest a sixfold increase in riverine N_2_O emissions from 1900 to 2016 across the Arctic region, which is much faster than the global average rate of increase. Among cryosphere regions, river N_2_O emissions show similar patterns to lakes: Continuous permafrost rivers are N_2_O sinks, while discontinuous and sporadic/isolated permafrost rivers are N_2_O sources of the atmosphere.

N_2_O emissions from northern cryosphere lakes and rivers total to 5.36 (−1.39 to 12.15) Gg N_2_O year^−1^. This represents ~1.5% of global inland waters N_2_O emission (*36*), far less than the water surface area proportion. Although permafrost soils harbor a large storage of nitrogen, bioavailable nitrogen is generally limited in permafrost environments due to strong plant-microbe competition (*61*). As such, aquatic systems in the cryosphere region also exhibit widespread nitrogen limitation (*62*–*64*), which in turn limits N_2_O production and outgassing (*26*, *29*). In addition, low temperatures in the cryosphere inland waters may also weaken denitrification and nitrification rates and impair N_2_O saturation status (*60*, *65*).

N_2_O measurements in the cryosphere inland waters are still sparse, which hampers accurate quantification of the cryosphere N_2_O budget. For instance, the northern glacial lakes and rivers have no N_2_O measurements available at the time of preparing this study. Although the overall magnitude of N_2_O emission from northern cryosphere inland waters plays a minimal role in global inland water N_2_O budgets (*36*), future warming may release more soil nitrogen and enhance N_2_O emissions (*26*). Permafrost soil can potentially release, export to, and concentrate the bioavailable nitrogen loading in inland waters under a warming climate (*61*), transforming these water bodies from minor N_2_O sources to N_2_O outgassing vents (*25*, *29*). The mechanisms underlying N_2_O sources or sinks in cryosphere inland waters are largely unknown and require further study.

### Inland water GHG emissions offset the terrestrial carbon sink

**Fig. 4.**
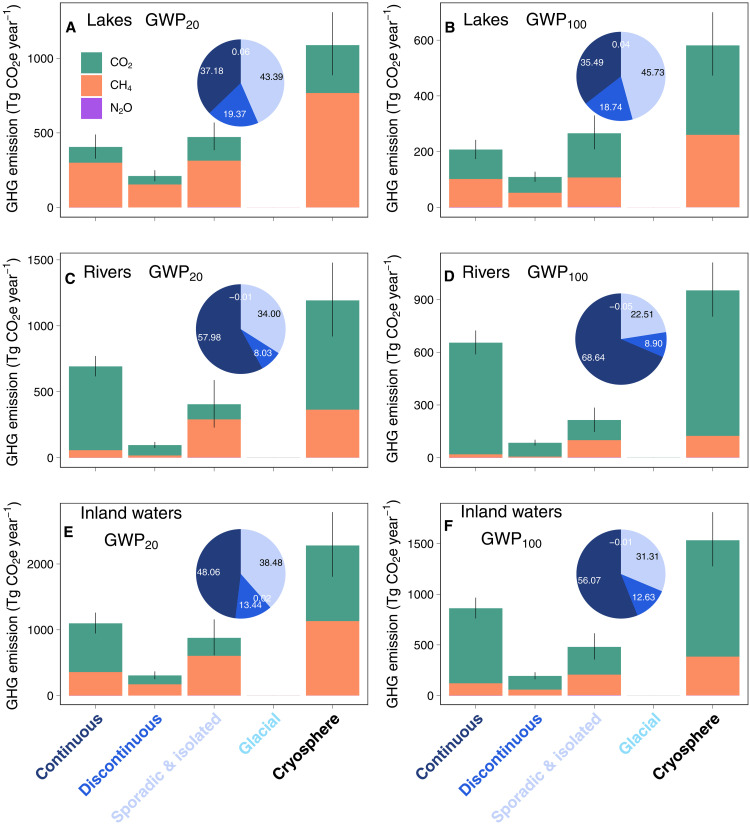
CO_2_e GHG emissions from northern cryosphere inland waters. (**A** to **F**) Median CO_2_e GHG emissions from lakes, rivers, and inland waters (lakes+rivers) using the GWP_20_ and GWP_100_ scenarios. N_2_O emissions are minor; therefore, the purple N_2_O emissions are not visible in the barplots. Bars indicate Q1 and Q3. The inlet pie charts in each subplot present the percentage portions of CO_2_e GHG emissions for each of the cryosphere zones (fill colors echo the color of the *x* axis labels for each cryosphere zonation). The contribution from glaciers is minor; therefore, the turquoise color is not visible in the pie charts.

Our syntheses shows that the northern cryosphere inland waters are globally important GHG sources, with estimated overall CO_2_e emissions of 2281 (1806 to 2788) or 1534 (1276 to 1811) Tg CO_2_e year^−1^ at GWP_20_ or GWP_100_, respectively (Fig. 4 and Table 1). The corresponding C and N emissions from northern cryosphere inland waters are 324 (282 to 370) Tg C year^−1^ (CO_2_-C+CH_4_-C, table S4) and 1.71 (−0.44 to 3.87) Gg N_2_O-N year^−1^, respectively. The CO_2_e GHG emissions account for 17 to 18% of global inland water GHG emissions (*36*). Cryosphere rivers have 64% higher CO_2_e GHG emissions than cryosphere lakes at GWP_100_ and 10% higher CO_2_e GHG emissions than lakes at GWP_20_, despite rivers being only one-fifth of the surface area of lakes (Fig. 1, B and C, and table S1). This suggests that the warming effect from rivers is greater than that from lakes in the cryosphere. Although lakes emit more than double the amount of CH_4_ compared to rivers, the large CO_2_ emissions in rivers make them the largest GHG contributors (Table 1 and table S3). In lakes, CH_4_ is the major contributor to GHG at GWP_20_, while CO_2_ surpasses CH_4_ in terms of CO_2_e GHG emissions at GWP_100_ (Fig. 4 and Table 1). The N_2_O emissions from inland waters are minor, collectively contributing 1.46 (−0.38 to 3.32) Tg CO_2_e year^−1^, less than 1% of the total CO_2_e GHG emissions. When comparing the total emissions from the four cryosphere zones, continuous permafrost region is clearly the largest inland water GHG source, followed by sporadic/isolated permafrost inland waters (Fig. 4, E and F), whereas glacial region are negligible sources. Seasonally, the variations of GHG emissions (fig. S10 and table S3) are caused by the collective effects of seasonal variations of open water surface areas and GHG flux rates. The organic matter decomposition rate and terrestrial-aquatic connectivity are enhanced when active layer thaws during the warm and rainy seasons (*66*, *67*). This could potentially elevate terrestrial inputs and internal production of inland water GHGs. The open water surface areas reach maximum during summer or autumn (Fig. 1, B and C, and table S1), which can overlap with high GHG flux rates. Consequently, the highest CO_2_e GHG emission from inland waters occurs in September (fig. S10 and table S3). We also tested upscaling of cryosphere lake GHG emissions using their size bins across months (see Materials and Methods). Although this size bin–based upscaling cannot be implemented into each cryosphere zone because not enough data for each size class, the results (tables S5 and S6) show distinct seasonal variations as for all lakes. The data gaps of GHGs toward the colder shoulder seasons (i.e., early spring, late autumn, and winter; see fig. S4) suggest that the GHG emissions from these seasons may have large uncertainty. More measurements are needed to improve the seasonal upscaling of GHG emissions from the northern cryosphere inland waters.

**Fig. 5.**
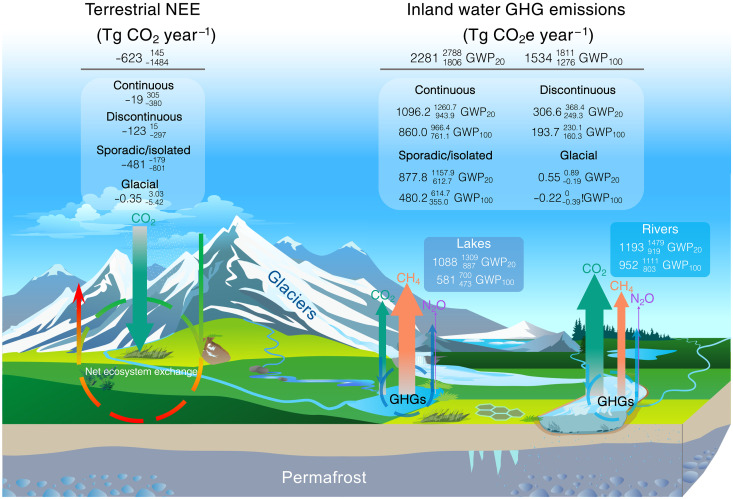
Inland water CO_2_e GHG emissions and land-atmospheric NEE of CO_2_ from the northern cryosphere. The inland water CO_2_e GHG emissions (Tg CO_2_e year^−1^) and terrestrial NEE (Tg CO_2_ year^−1^) are expressed as ${\text{median}}_{Q1}^{Q3}$ and summarized by cryosphere zones. The size of the arrows for each GHG is proportional to their respective emission magnitudes. The annual NEE of the northern cryosphere region are derived from the SPL4CMDL (*68*) dataset.

Using a gridded monthly NEE product (*68*), we can compare the monthly and annual terrestrial NEE with inland water GHG emissions in the cryosphere (fig. S11). We find that most of the monthly inland water GHG emissions are much lower than the absolute value of NEE, except that the September NEE is lower than inland water GHG emissions. However, the annual inland water GHG emissions exceed the annual NEE when summing up the NEE from all months. More specifically, annual median CO_2_e GHG emissions (GWP_100_) from inland waters are 146% higher than the median terrestrial NEE from SPL4CMDL [−623 (−1484 to 145) Tg CO_2_ year^−1^] for the entire cryosphere zone (Fig. 5 and fig. S11). If we compare the CO_2_e GHG emissions under GWP_20_, inland water median GHG emissions are 266% higher than the NEE (table S4). When we only consider the carbon mass (CO_2_-C and CH_4_-C) emissions for inland waters rather than CO_2_e under the GWP, the magnitude of terrestrial NEE is still considerably lower than the inland water carbon emissions (table S4).This suggests that the terrestrial carbon sink would be substantially offset by inland water GHG emissions in the cryosphere on an annual basis. However, it is important to note that other carbon fluxes are not accounted for in this comparison. For example, we do not consider potential C sinks in inland waters such as carbon burial or lateral exchange. Furthermore, the NEE is based on CO_2_ measurements and we do not know the contribution from CH_4_ and N_2_O ecosystem exchange in the terrestrial cryosphere. Last, we assume consistent annual inland water GHG emissions over the study period (1983 to 2022), whereas the terrestrial carbon sink has likely increased over time. While the comparison of terrestrial NEE and inland water CO_2_e GHG emissions is incomplete in terms of a GHG budget, it shows the relative importance of inland water bodies in the cryosphere land-aquatic continuum. We find that the continuous permafrost zone is responsible for most of the NEE offsets by CO_2_e GHG emissions showing 841 Tg CO_2_e year^−1^ higher GHGs than the absolute NEE (Fig. 5 and fig. S11). Our findings, based on a broad spatial scale and multiyear median annual NEE analysis, highlight the significance of inland water GHG emissions in offsetting the terrestrial carbon sink.

### Uncertainties in the upscaling and implications for future research

Our estimates of GHG emissions from cryosphere inland waters in this study is built on a synthesis of an unprecedentedly large spatiotemporally resolved GHG dataset, including more observations not reported in previous efforts (*13*, *15*, *47*, *69*). Our upscaling incorporates both the spatial and temporal variability of areal GHG fluxes by bootstrapping the monthly GHG fluxes in separate cryosphere zones. We also use monthly resolved open water surface areas along with statistics of monthly flux rates, which avoids biased upscaling using simple average flux rates and static surface areas. These efforts substantially reduced the spatiotemporal uncertainties associated with areal flux rates and surface area for the upscaling. However, our estimates do not come without shortcomings. Cryospheric lakes can accumulate large amounts of GHGs during the winter season, which are evaded during the short ice break-up period (*47*–*49*). Our datasets include limited ice-melt flux data during the ice-out period (fig. S4), implying that our calculated annual GHG emissions are likely underestimated. Because we use the minimum bootstrapping statistics of the year to substitute the months with 0 to 3 monthly measurements of the dataset (see Materials and Methods), our GHG emission estimate may be considered conservative.

Given the ongoing climatic crisis, future GHG emissions from cryosphere inland waters will likely undergo substantial changes. Yet, we are uncertain about the direction of change for each GHG because we do not fully understand the controls and mechanism of GHG emissions in the cryosphere. However, previous research has shown that warming climate directly liberates more thawed organic matter from permafrost or ice sheets to the aquatic networks (*11*). The intensifying water cycles under warming (*70*) can also route more terrestrial carbon into the aquatic network through runoff (*22*). Such increased carbon and nitrogen loadings in inland waters, along with expected higher mineralization, methanogenesis, or denitrification rates under warming (*65*, *71*, *72*), will likely lead to enhanced GHG fluxes per unit area. In addition, changing ice phenology and waterscape extents also affect the inland water GHG emissions. The shortened ice-cover season under warming lengthens the duration of GHG outgassing. The widening of rivers in the Arctic and QTP (*73*) can potentially create higher connectivity between aquatic and terrestrial ecosystems, thereby enhancing GHG emissions. Lake open water surface area is found to diminish in the Arctic caused by widespread draining (*74*), whereas a rapid expansion of lake areas was observed in the QTP (*75*). Draining lakes creates more aerobic environments that restrict CH_4_ emission but may enhance CO_2_ emission, while the opposite, expanding lakes enhance CH_4_ emission but may restrict CO_2_ emission, which may also be true. These contrasting trends and processes complicate the prediction of the overall and individual forms of cryosphere inland water GHG emissions. The collective effects of warming and degradation of the cryosphere on inland water GHG emissions require further investigations that consider both the complex changes in water body extent and the mechanistic controls of GHG dynamics. Furthermore, it is important to note that there are considerable gaps in GHG observations across large areas of the northern cryosphere (Fig. 1A and fig. S2). Regrettably, we have observed a decline in GHG measurements specifically in northern cryosphere inland waters since 2015–2016 (fig. S3). Going forward, it is crucial to enhance GHG monitoring efforts in less represented regions (*76*) such as Siberia, Northern Canada, northwest QTP, and all glacier regions. Addressing these data gap depends on both domestic efforts and international collaborations from the pan-cryosphere regions. It is also essential to strengthen observations during the shoulder seasons to capture a more comprehensive understanding of GHG seasonality. Further research should investigate if the pronounced but inconsistent seasonal pattern found for the three GHGs in our synthesis is a general trend or due to the limited data. Meanwhile, advanced aquatic GHG sensors, remote sensing, and machine learning should also be exploited to improve inland water GHG estimates.

Our study suggests that the warming effect from inland water GHG emissions substantially offset the cooling effect from land carbon uptake. While our findings may be unexpected at first, we find two main explanations for this. First, the long nongrowing winter season largely reduces the annual land carbon sink (*77*), while cryosphere inland water ecosystems are net-heterotrophic in nature (*53*) and act as large GHG sources almost all year round. Second, sizable (but not all) inland water GHG emissions may decouple from the land-atmosphere carbon exchange because of the “time lag” between the inland water and terrestrial GHG cycles (*38*). For example, the CO_2_ and CH_4_ emitted by permafrost thaw–affected inland waters in contemporary times may be sourced from ancient carbon assimilated thousands of years ago (*16*, *17*, *78*). As such, we argue that the biogeochemical processes of GHG in cryospheric inland waters are partially interconnected with yet partially decoupled with terrestrial processes. The linkages primarily occur through organic matter input, nutrient supply, and hydrological connectivity. However, the decoupling arises due to differences in biogeochemical cycle timescales. The magnitude of land carbon sink across the northern region is still under debate (*79*, *80*). Despite uncertainties in our cryosphere GHG emissions and caveats concerning the comparison with terrestrial NEE, our synthesis highlights the important role of inland waters in the terrestrial-aquatic continuum within a watershed boundary in the northern cryosphere.

To summarize, our study shows that northern cryosphere inland waters are important GHG emitters, with CO_2_ and CH_4_ dominating the CO_2_e GHG emissions. N_2_O emissions are currently negligible in total CO_2_e GHG emissions but may increase in a warming cryosphere in the future. We find GHG emissions show strong seasonality, which is caused by seasonal changes in water surface area and flux rates. Overall, cryosphere rivers emit more CO_2_e GHGs than cryosphere lakes. The annual inland water GHG emissions are found to exceed the NEE, highlighting the importance of aquatic GHG emissions in offsetting the terrestrial carbon sink but also signaling some extent of decoupling between inland water and terrestrial GHG biogeochemical cycles. The changes in inland waters under a diminish cryosphere may have substantial impacts on the aquatic GHG emissions. More actions are needed to fully understand and predict the effects of degrading cryosphere on the inland water GHG budget as the cryosphere is crucial to achieving climate goals.

## MATERIALS AND METHODS

### GHG flux data synthesis

We compiled available GHG concentration and flux observations to curate a spatial and temporally resolved Cryosphere Inland Water Greenhouse Gases Database (or CIWD-GHG) (*43*). Peer-reviewed papers, dissertations, and theses published before April 2023 were searched using Web of Science, Google Scholar, ProQuest Dissertations & Theses Global, and China National Knowledge Infrastructure. We also searched public data repositories, including the Arctic Data Center, Zenodo, Environmental Data Initiative, and PANGAEA, to include relevant datasets. The following search strings were used: (methane OR CH_4_ OR carbon dioxide OR CO_2_ OR nitrous oxide OR N_2_O OR greenhouse*) AND (river OR stream OR lake OR pond OR reservoir) AND (Arctic* OR Tibet* OR Greenland OR Antarctic OR glacier* OR permafrost). We conducted the data source search multiple times before April 2023 to ensure completeness. We screened and selected the searched results following a consistent criterion: The GHG concentrations or fluxes should be measured in permafrost-borne or glacier-borne inland water systems, including streams, rivers, ponds, lakes, and reservoirs. Specifically, the cryosphere extent in this study was defined using the currently available glacier extent (*2*) and permafrost extent maps (*1*). We used the boundaries defined within these previous maps (*1*, *2*) to divide the cryosphere into four zonations: continuous permafrost, discontinuous permafrost, sporadic/isolated permafrost, and glacial regions. Different permafrost types were formed under joint controls of climate, geography, hydrology, vegetation, and topography factors that represent the natural gradient of the permafrost-affected environment. Differences in permafrost types can inevitably affect the properties of inland water within those permafrost zones. Comparing GHGs under different permafrost types can also give insight into potential impacts of changes under permafrost conditions with warming. We therefore compare inland water GHGs in different permafrost types. Study sites outside these cryosphere extents were excluded. Wetland ecosystems were not considered in this synthesis because they have distinct characteristics compared with inland waters. The inland waters in northern cold regions with no glaciers or any type of permafrost distribution were also excluded. Floodplain lakes connected with river channels were excluded; however, if the lakes barely connected with (high closure) river channels, then they were included as lake sites. Gas concentration data in permanently ice-covered water bodies were not included. The main GHG data include CO_2_ concentration, CH_4_ concentration, N_2_O concentration, CO_2_ flux, diffusive CH_4_ flux, ebullitive CH_4_ flux, total CH_4_ flux, and N_2_O flux. Auxiliary data, such as water body physical and chemical characteristics, cryosphere zonation, climate, and land cover, were also collected where available. We compiled the lake dataset (including lakes, ponds, and reservoirs) and river dataset (including rivers and streams) separately. For the river dataset, we directly extract some of the cryosphere river data from GRiMeDB (*81*). Because GRiMeDB is a CH_4_-dominant dataset, we performed additional searches on CO_2_ and N_2_O studies and added more data published since the cutoff date of GRiMeDB. We also screened the northern aquatic methane datasets by Kuhn *et al.* (*69*) and Wik *et al.* (*13*) for completeness.

The dataset was organized following the example established by Stanley *et al.* (*81*) as we aim to compile a spatial and temporally resolved dataset. Each of the river and lake data files consisted of four subtables: a source table with the data sources, a site table with site information from each source, a concentration table with concentration data, and a flux table with flux data. All subtables were linked with unique source IDs, while the sites, concentration, and flux tables were also linked with unique site IDs. The shortest temporal resolution was daily, and sub-daily measurements were averaged to daily data, while data reported in monthly, seasonal, and annual scales were also included. The spatial resolution was usually at the plot scale, while aggregated sites were also included. All the detailed spatiotemporal information of the data measured was entered into the data table for further analysis. We do not have a uniform standard for dividing the seasons because of regional heterogeneity. Seasons were assigned according to the site description for each study.

The largest reported CO_2_ and CH_4_ flux values were 86,827 and 6100 mmol m^−2^ day^−1^, respectively (*82*, *83*). To avoid possible distortion of extreme flux values, we removed a few outliers using Rosner’s test before data analyses. This step kept CO_2_ and CH_4_ flux values smaller than 86,827 and 2400 mmol m^−2^ day^−1^, respectively, which removed three extremely large CH_4_ flux values from the lake dataset and one extremely large CO_2_ flux value from the river dataset. N_2_O data had no apparent extreme values.

Notably, previous regional and global data synthesis efforts including Wik *et al.* (*13*), Kuhn *et al.* (*69*), Stanley *et al.* (*57*, *81*), and Rosentreter *et al.* (*58*) have improved our knowledge of GHG emissions from inland waters. For the cryosphere, these datasets either focused on incomplete GHG species and water bodies or had limited spatial coverage. Our CIWD-GHG dataset is, to our knowledge, the most comprehensive global cryosphere inland water GHG dataset to date. The finalized dataset includes 1585 lake sites (9225 flux data records) and 1390 river sites (3205 flux data records) (fig. S1). Although our datasets include dozens of sites in South America and Antarctica, these data were excluded from the analyses due to limited GHG measurements and the absence of surface area for inland waters in Antarctica.

### Monthly inland water surface area

The monthly resolved inland water surface area is the foundation for accurate GHG emission upscaling. This is especially important for cryospheric inland waters with drastic seasonal water-level fluctuations and ice phenology. We used a global monthly water surface area product (*45*) for the open water surface area of lakes and reservoirs larger than 0.1 km^2^. Zhao *et al.* (*45*) reconstructed the monthly open water area by considering both the ice duration and water-level fluctuation through seasons. The dataset contains 1.42 million lakes across the globe, with the open water surface area of each lake of the HydroLAKES framework (*84*) available from 1985 to 2018. We selected the cryosphere lakes by intersecting the HydroLAKES shapefiles with the northern cryosphere extent, yielding 971,713 lakes within the northern cryosphere extent. However, 177,028 of these lakes have a maximum monthly area smaller than 0.1 km^2^, which is larger than the minimum lake size defined by HydroLAKES. This can be attributed to the mixed-pixel uncertainty for small lakes when using 30 m in resolution satellite images to extract water surfaces (*45*). Thus, we truncated the 177,028 lakes with a maximum monthly area smaller than 0.1 km^2^ and calculated the surface area for the remaining 794,685 lakes (56% of HydroLAKES) of every month by averaging the data from the past two decades (2000 to 2018) to obtain near-present monthly open water surface area. The total monthly open water surface area of the northern cryosphere lakes larger than 0.1 km^2^ ranges from 174 to 986,984 km^2^ (Fig. 1, A and B, and table S1).

Unfortunately, seasonal surface area data for lakes and reservoirs smaller than 0.1 km^2^ are currently not available. The available static small lake area data products also show large discrepancies. The widely used GLOWABO database (*85*) is likely to overestimate the area of small lakes owing to the inclusion of nonlake polygons. Statistical extrapolation-based global estimates by Downing *et al.* (*86*) may also exaggerate small lake areas because small lake areas and abundance distribution were divergent from the Pareto law in many nonflat regions (*87*–*89*). A recent global lake mapping GLAKES (*20*) provided reliable static estimates of lake area of >0.03 km^2^. We used this product to extract the lake area of the size class 0.03 to 0.1 km^2^, which is 66,056 km^2^ for 1,202,851 lakes.

The area and abundance of smaller lakes (0.0001 to 0.03 km^2^) were extrapolated with a log-log linear regression (fig. S9). Ponds smaller than 0.0001 km^2^ are not considered in this analysis because they contribute a negligible total lake area (*90*). We first masked the GLAKES within the northern cryosphere extent and grouped all the lake areas with a bin size of 0.0001 km^2^. The average area for each bin was calculated based on the lakes included in that bin. The lake number in each bin was counted. Then, we selected the average area between 0.03 and 0.1 km^2^ and fitted a log-log linear regression between the average area and lake number of each size bin (number of lakes in each bin = 23.74398 × average bin area^−1.490131^, *R*^2^ = 0.9965; fig. S12). Using this regression relationship, we calculated the number of lakes in each bin with the average bin area starting from 0.0001 to 0.03 km^2^ on a 0.0001-km^2^ interval. The total extrapolated lake area can be derived by summing the multiplication of the number of lakes in each bin and the average bin area. We estimated that the total cryosphere lake number in the size range of 0.0001 to 0.03 km^2^ is 54,797,691 with a total area of 74,898 km^2^. Therefore, the cumulative lake area between the size classes 0.0001 and 0.1 km^2^ is estimated as 141,018 km^2^. We treated this area as the maximum area of the year for the size class 0.0001 to 0.1 km^2^ and assumed that this maximum area also occurs in September as the lakes with size class 0.1 to 1 km^2^. Then, we multiplied the ratios of the monthly lake area to the lake area in September and extrapolated the monthly areas for the smallest 56 million lakes (i.e., 0.0001 to 0.1 km^2^). The final monthly lake open water surface area with a static size larger than 0.0001 km^2^ ranges from 293 to 1,127,842 km^2^ (Fig. 1A and table S1). Our estimated area for small lakes (141,018 km^2^ in the size bin 0.0001 to 0.1 km^2^) accounts for 12.5% of the total lake area, closely aligning with the 11.5% proportion for the same size bin in a recent estimation of pan-Arctic lake areas based on high-resolution remote sensing (*91*). This suggests that our extrapolation closely approximates the actual abundance and overall area of small lakes. Note that clipping of a georeferenced global dam and reservoir dataset (*92*) by the cryosphere extent shows no reservoirs smaller than or equal to 0.1 km^2^ in the cryosphere, while the cryospheric reservoirs larger than 0.1 km^2^ (0.02% of total cryosphere lakes and reservoirs) have been included in the monthly lake area data (*45*) we used.

The current widely used global river surface area data (*21*, *55*, *93*) were resolved on an annual basis, except the monthly river surface data created by Liu *et al.* (*22*). This estimation was completed with coupled downstream hydraulic geometry, at-a-station hydraulic geometry, and an improved watershed hydrology-based land surface area segregation scheme. The derived monthly surface area included the smallest streams starting with a width of 0.3 m. Both ephemeral and ice-covered extents were estimated to derive monthly open water surface areas. These surface data were produced under the HydroBASIN level 4 framework (*94*). We used the dataset to subset the monthly cryospheric river and stream open water surface area. Because the river surface area is given for each HydroBASIN level 4 basin, some of the basins are partially underlined by cryosphere extent. We calculated the river surface area of such basins by multiplying the cryosphere percentage coverage by the total river surface area of that basin. This yielded a monthly river surface area of 3021 to 200,722 km^2^ (Fig. 1B and table S1), which is marginally larger than the previously estimated Arctic’s monthly river surface area (*22*), probably because we incorporated the QTP rivers. We assumed that all the cryosphere rivers should be completely frozen during the coldest months. Thus, the smallest monthly river surface area (January) of each cryosphere zone was subtracted from each month. The final monthly cryosphere river surface area ranges from 0 to 198,840 km^2^ (fig. S8 and table S1).

### Upscaling

For each of the water body types, GHGs, cryosphere zones, and months, a multilevel bootstrapping method (fig. S13) was performed to estimate GHG fluxes (*95*) from northern inland waters. We used GHG fluxes per unit area for upscaling, excluding concentration values to avoid the added uncertainty from gas transfer velocity. We performed 10,000 iterations and used the means of each group of samples segregated by two water body types, four cryosphere zones, 12 months, and three GHG types. We used means over medians in the bootstrapping because the extreme values have been removed to avoid distortion and mean values can more accurately account for high values that cannot be omitted from the observations (*13*). We used the medians of generated mean value distributions for the upscaling and Q1 and Q3 as uncertainty ranges. If a specific month of the cryosphere region had fewer than three measurements, then we discarded the original bootstrap statistics and applied the lowest absolute statistics from other months to this month in this region to reduce the uncertainties (fig. S13). The GHG emissions from cryosphere inland waters were upscaled each month for four cryosphere zones (continuous permafrost, discontinuous permafrost, sporadic and isolated patches of permafrost, and glacial) by multiplying the respective open water surface area and bootstrapped flux rates. Then, we derived the monthly emission as the sum of emissions from all cryosphere zonations and annual emissions as the sum of monthly emissions. Our upscaling did not differentiate lakes and reservoirs because reservoirs only account for a small portion (183 of 794,685, or 0.02%) of the cryosphere lakes and reservoirs.

### CO_2_e GHG emissions

CO_2_e GHG emissions were calculated to compare the relative radiative forcing of CO_2_, CH_4_, and N_2_O following the GWP_20_ and GWP_100_ time horizons based on the IPCC Sixth Assessment Report (*96*). Specifically, under GWP_20_, 1 kg of CH_4_ or N_2_O has the same GWP as 79.7 or 273 kg of CO_2_, respectively. For GWP_100_, 1 kg of CH_4_ or N_2_O has the same GWP as 27.0 or 273 kg of CO_2_, respectively. The overall CO_2_e GHG emissions of the three gases were derived by summing the CO_2_e emissions of individual gases, cryosphere zonations, water body types, and months at respective time horizons (GWP_20_ or GWP_100_).

### Statistics

Wilcoxon test was used to test the difference of the inland water GHG flux rates between different cryosphere zones of the northern hemisphere. We considered the differences to be statistically significant when *P* < 0.05.

### NEE data sources and processing

To compare the inland water CO_2_e GHG emissions with the terrestrial carbon sinks, we considered two NEE datasets: the SMAP L4 Global Daily 9 km EASE-Grid Carbon Net Ecosystem Exchange, version 7 product (*68*) (SPL4CMDL) and a terrestrial net ecosystem exchange dataset inferred from the ACOS GOSAT v9 XCO2 retrievals (GCAS2021) (*97*). Although both datasets were used for assessing land carbon sinks at regional or global scales (*14*, *98*), the GCAS2021 data (*97*) may include CO_2_ exchanges from small inland waters and cause a double counting problem when compared with inland water GHG emissions. Therefore, we selected the SPL4CMDL dataset to compute monthly and annual NEE magnitudes from different cryosphere zones. This dataset provides global gridded (9 × 9 km) estimates of daily land-atmosphere NEE from 31 March 2015 to the present day, while the aquatic system is not included in the dataset (*68*). We downloaded 8 years of daily NEE data from 1 April 2015 to 31 March 2022 (2922 days) in GeoTIFF format from https://nsidc.org/data/spl4cmdl/versions/7. Then, the daily NEE files were imported in R and computed as monthly GeoTIFF files (fig. S14). Next, we extracted the monthly NEE with different cryosphere zone polygons defined earlier using the “exact_extract” function in the R package “exactextractr” (*99*). This function can compute the fraction of each raster cell that is partially covered by a polygon, which yields accurate extraction results. We calculated the NEE across different cryosphere zones by summing up the multiplication of each cell area (81 km^2^) with the NEE values from each cell of the respective cryosphere polygons. The monthly and annual median NEE and interquartile ranges (IQRs) were aggregated for each cryosphere zone and the entire cryosphere zone.

### Uncertainty analysis

The uncertainties associated with monthly areal GHG flux rates among each cryosphere zone were estimated using a Monte Carlo simulation that was implemented in the multilevel bootstrapping method. We generated a suite of statistics including the median, IQRs, and 95% confidence intervals of the datasets from the bootstrapping procedures. Because the GHG fluxes were non-normally distributed, we combined the median and IQR of each subgroup to propagate the grouped median and IQR as in (*95*).
