# Supplementary material for: Inland water greenhouse gas emissions offset the terrestrial carbon sink in the northern cryosphere
Source: Sci Adv. 2024 Sep 27;10(39):eadp0024. doi: 10.1126/sciadv.adp0024 (PMC11430465; doi:10.1126/sciadv.adp0024)
Supplement: Supplementary file 1 — Figs. S1 to S14 Tables S1 to S6 [file sciadv.adp0024_sm.pdf]

Science Advances
AAAS

Supplementary Materials for

Inland water greenhouse gas emissions offset the terrestrial carbon sink in the northern cryosphere

Chunlin Song *et al.*

Corresponding author: Chunlin Song, songchunlin@scu.edu.cn; Genxu Wang, wanggx@scu.edu.cn

Sci. Adv. **10**, eadp0024 (2024)
DOI: 10.1126/sciadv.adp0024

This PDF file includes:

Figs. S1 to S14
Tables S1 to S6

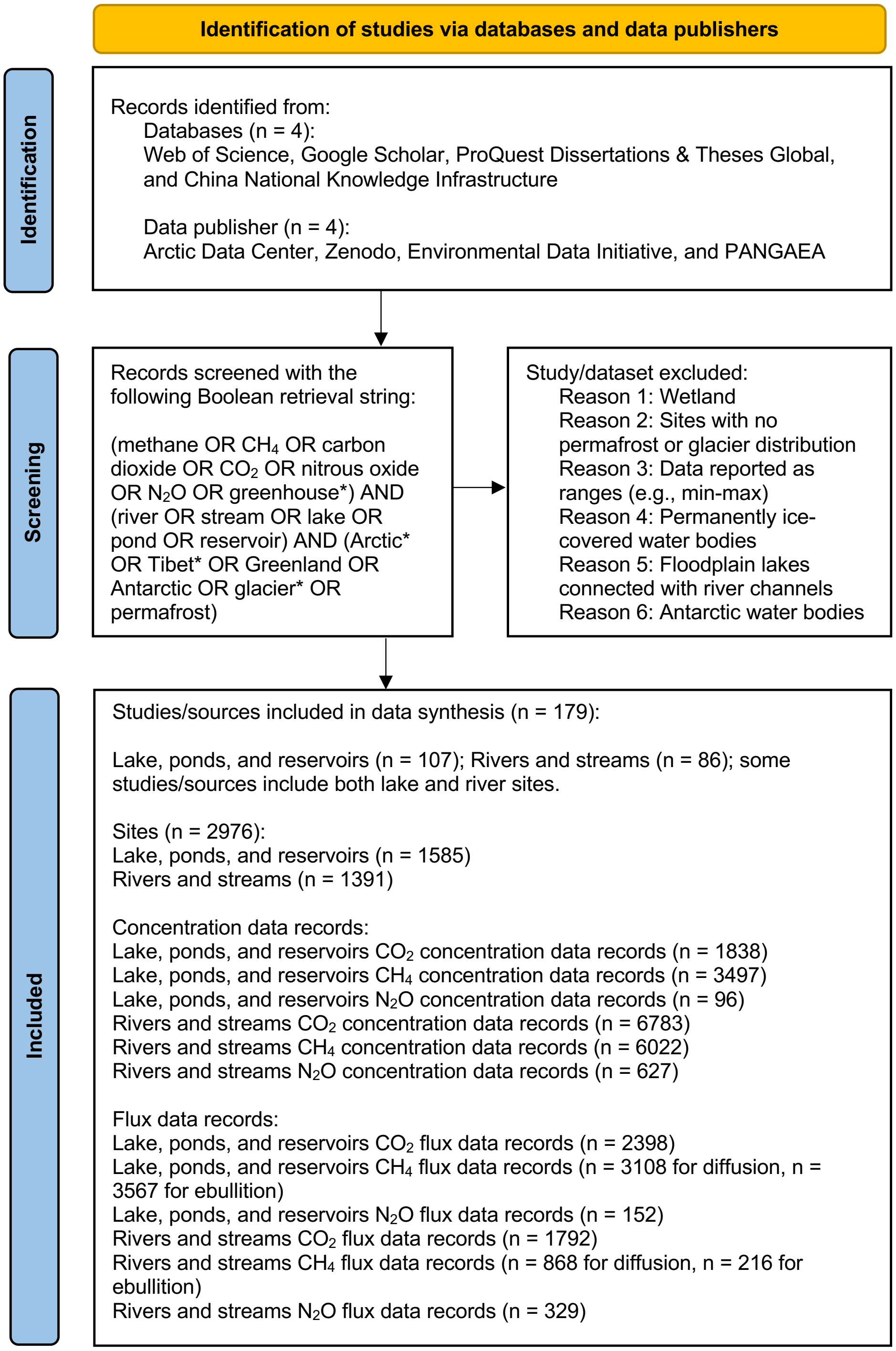

Fig. S1. PRISMA flow diagram for systematic reviews of GHGs in northern cryosphere inland waters. Modified from: Page MJ, McKenzie JE, Bossuyt PM, Boutron I, Hoffmann TC, Mulrow CD, et al. The PRISMA 2020 statement: an updated guideline for reporting systematic reviews. BMJ 2021;372:n71. doi: 10.1136/bmj.n71. For more information, visit: http://www.prisma-statement.org/

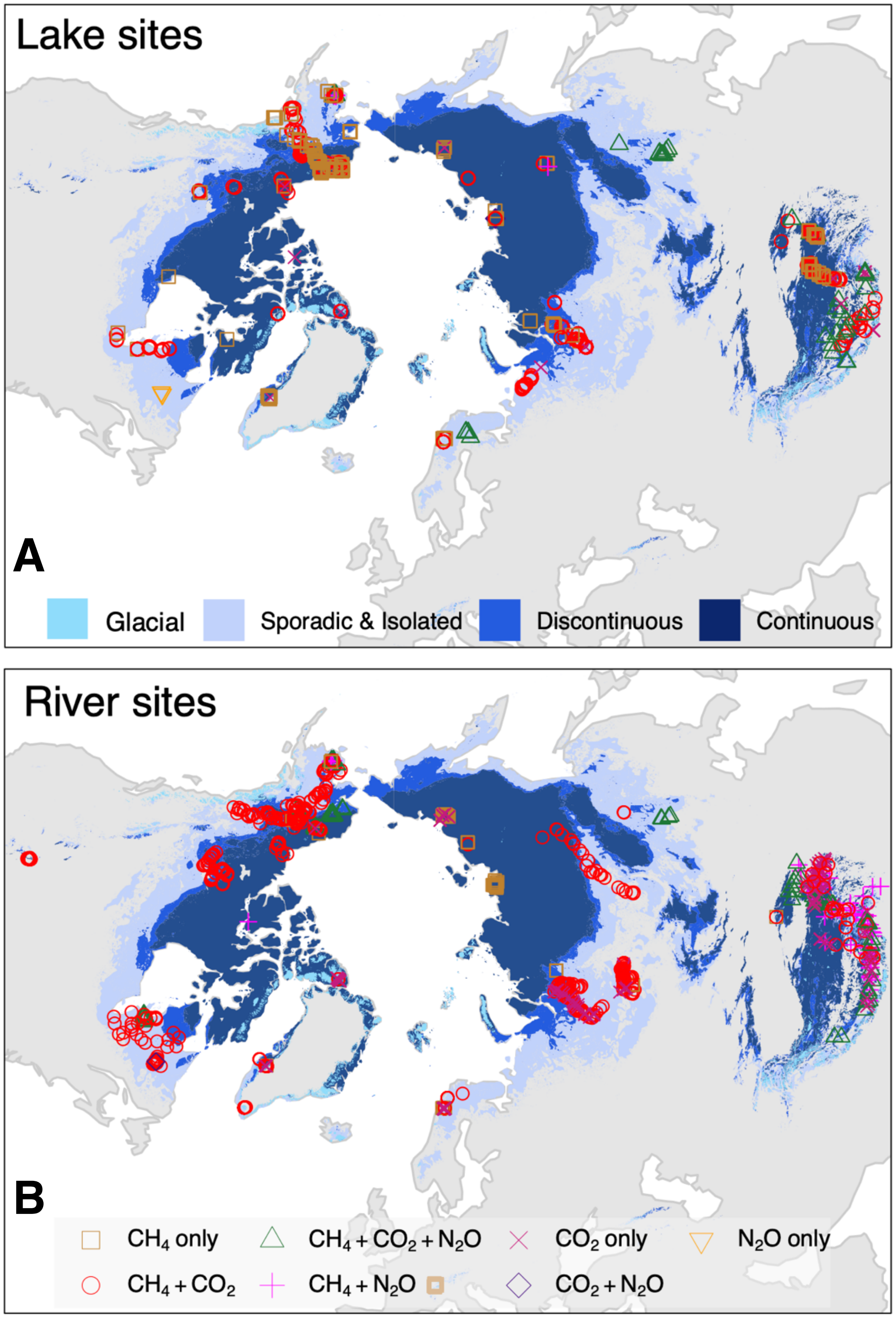

Fig. S2. **Maps of GHG observation sites of lakes (A) and rivers (B)**. Different point shapes denote the type of GHG measured at each site. Base map showing the distribution of permafrost and glacier of the northern hemisphere.

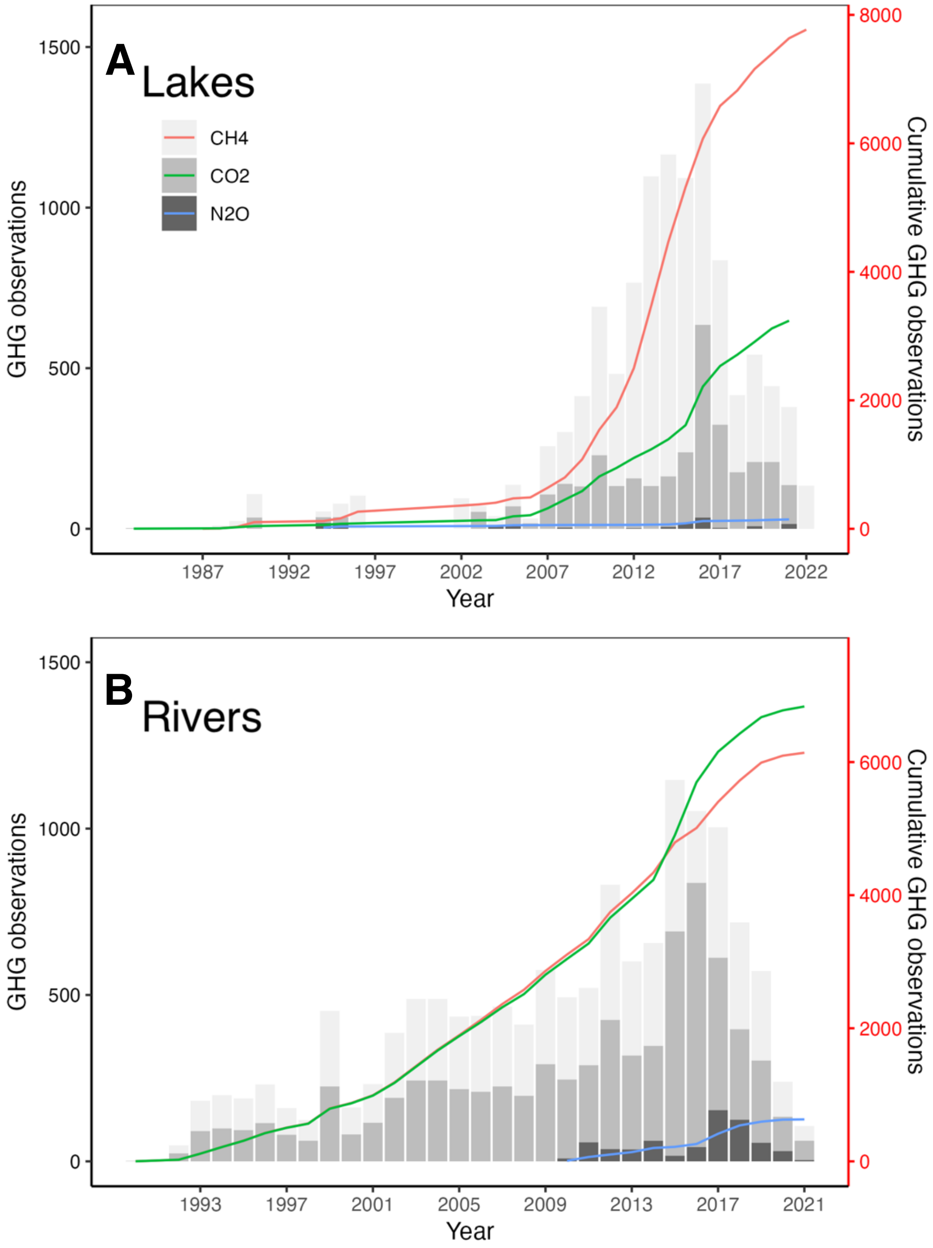

Fig. S3. Annual (bars) and cumulative (lines) GHG observation counts, either flux or concentrations, for lakes (A) and rivers (B) by year.

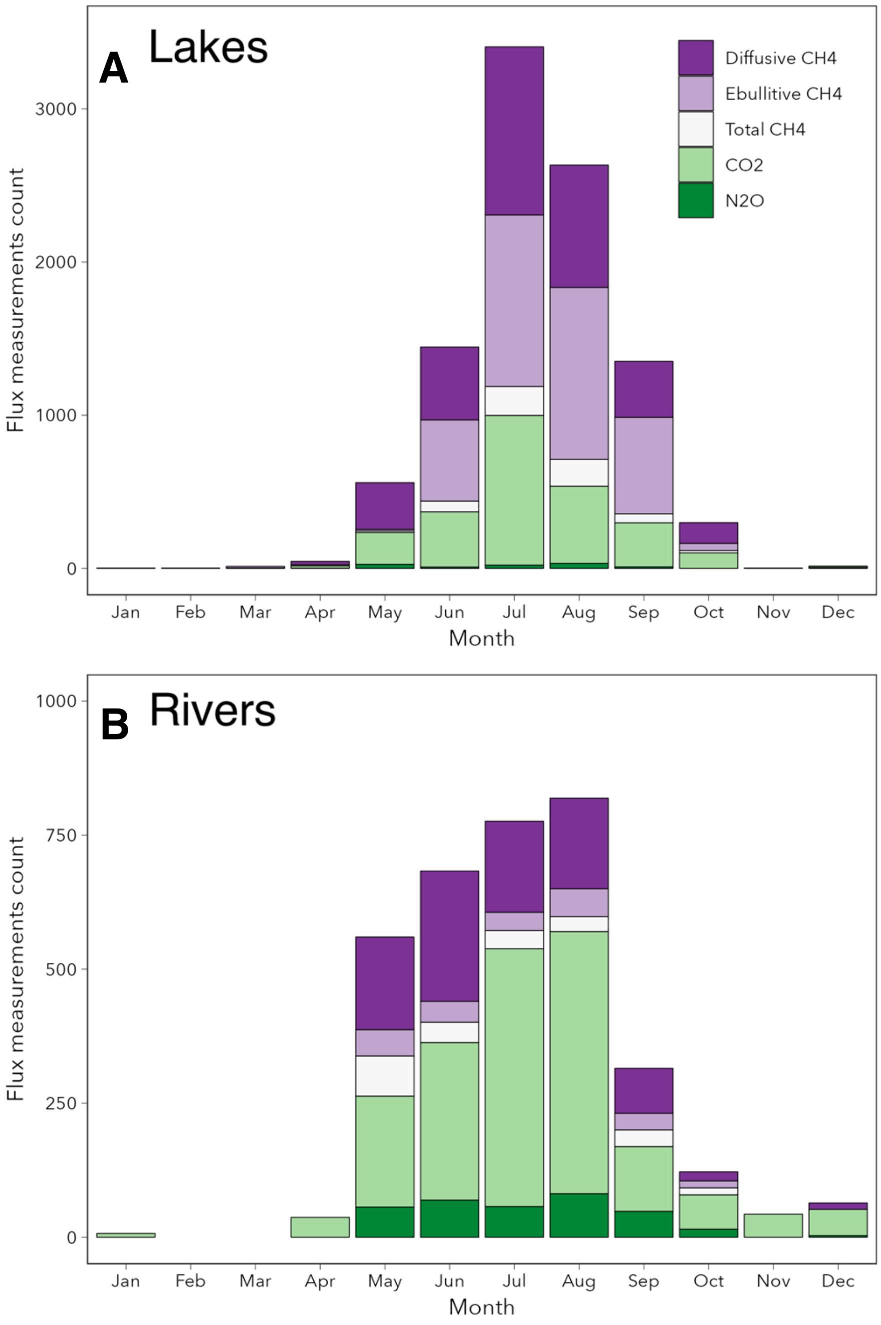

Fig. S4. GHG flux measurements count by months for lakes (A) and rivers (B).

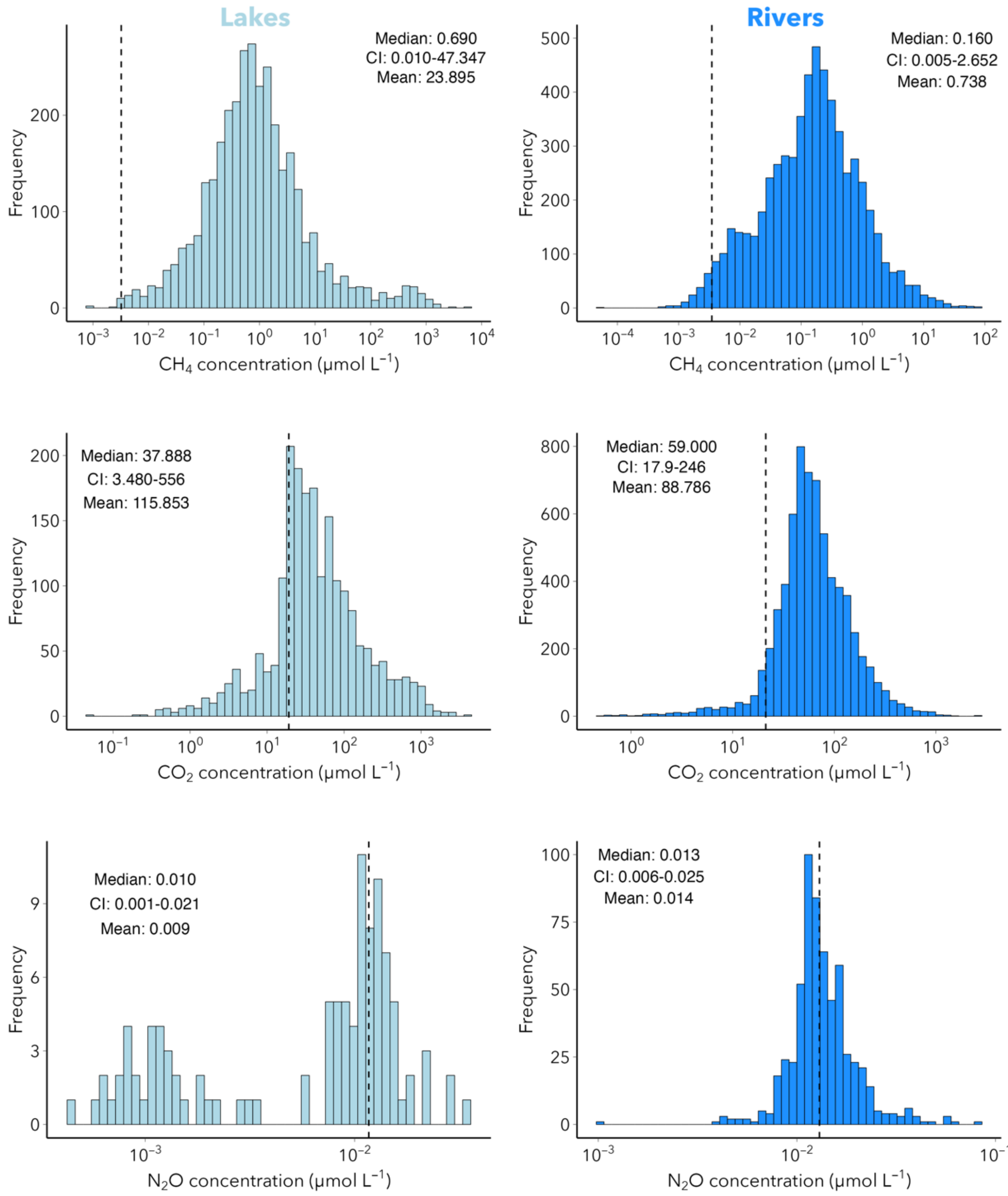

Fig. S5. Distribution of GHGs concentrations for cryosphere inland waters. Vertical dash lines are saturated dissolved CH_4 (0.0032 μmol L^{-1}), CO_2 (19.165 μmol L^{-1}), and N_2O (0.0116 μmol L^{-1}) concentrations with respect to the average atmospheric background concentrations.

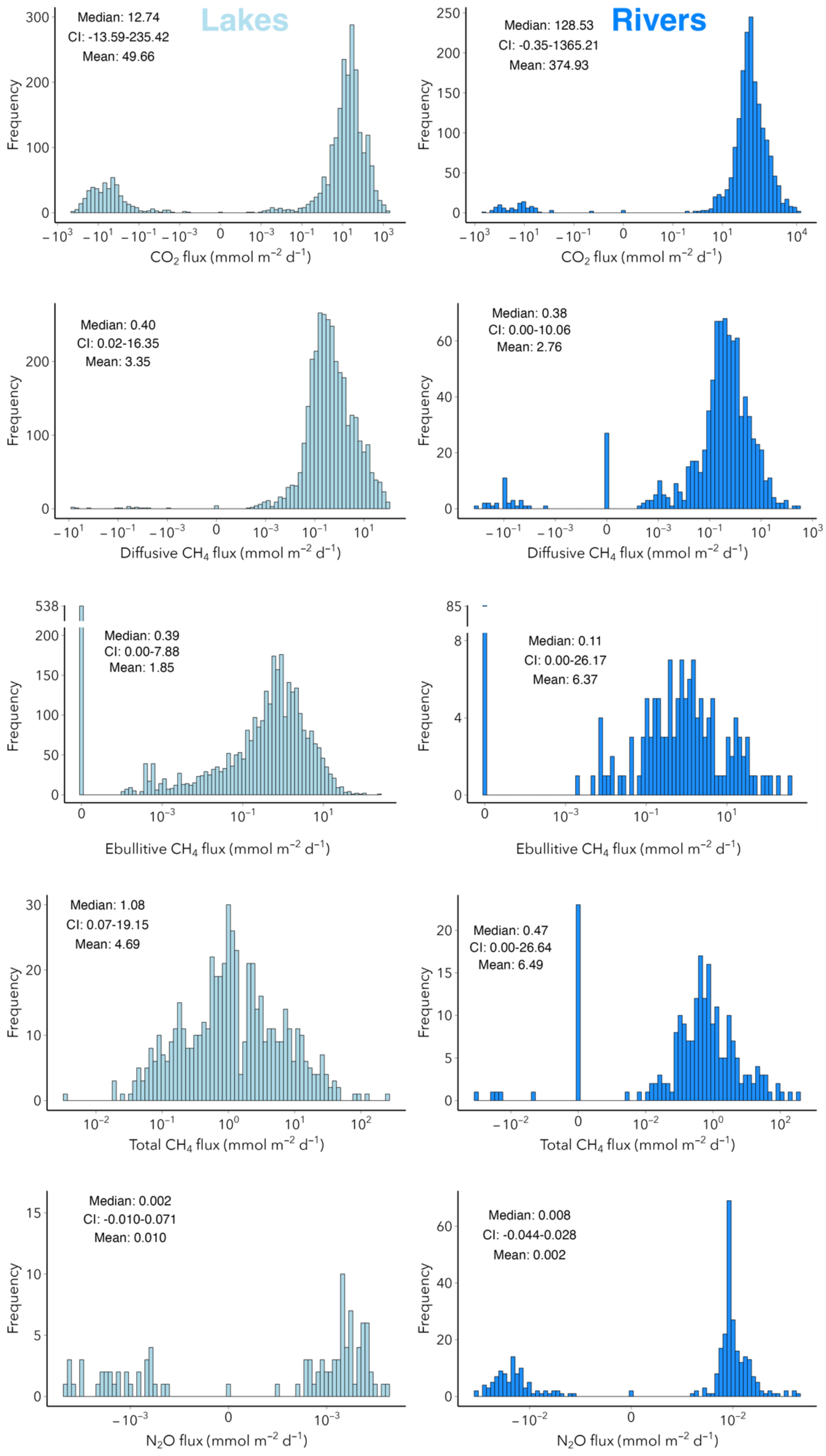

Fig. S6. Distribution of GHGs areal fluxes for lakes and rivers.

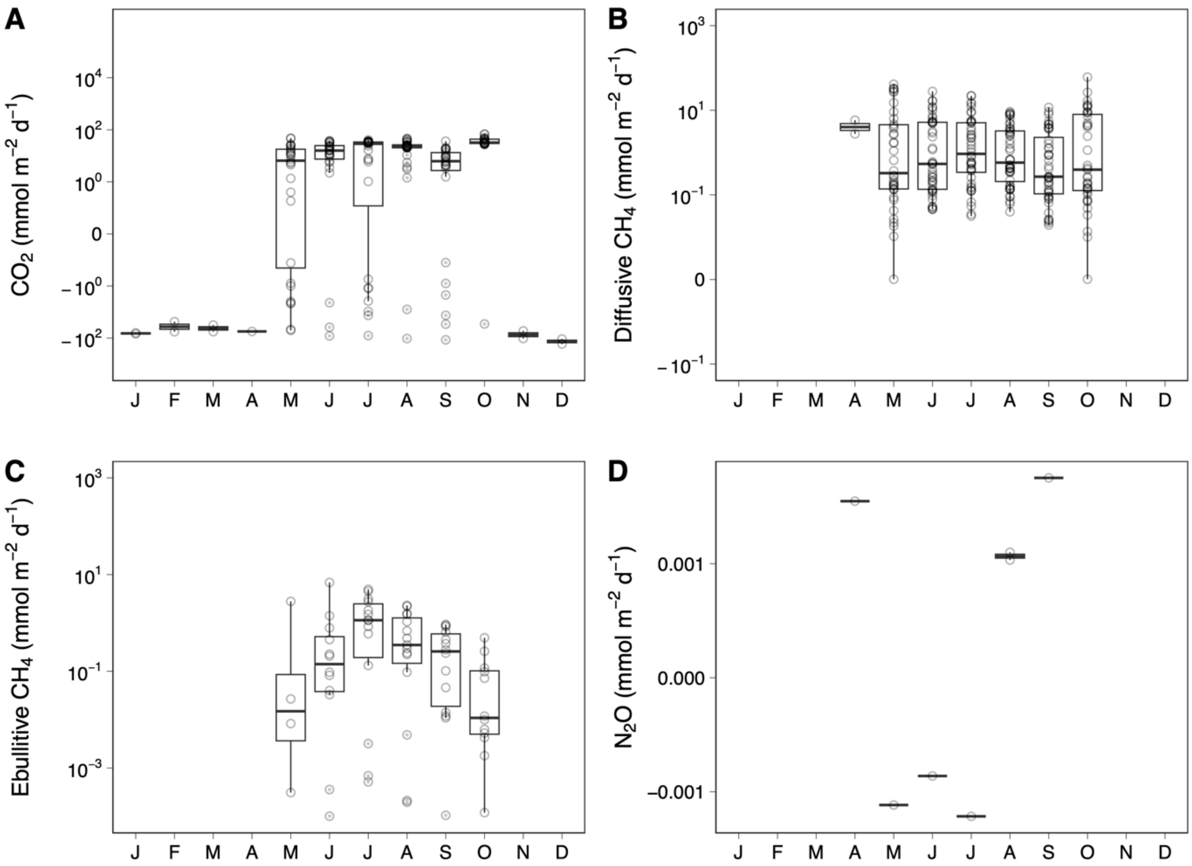

Fig. S7. **Seasonal variations of GHG flux rates in lakes using subsets of sites.** Sites with less than 5 months of observations were excluded in the analyses. Note that the y-axes for CO_2 and CH_4 fluxes are log-transformed.

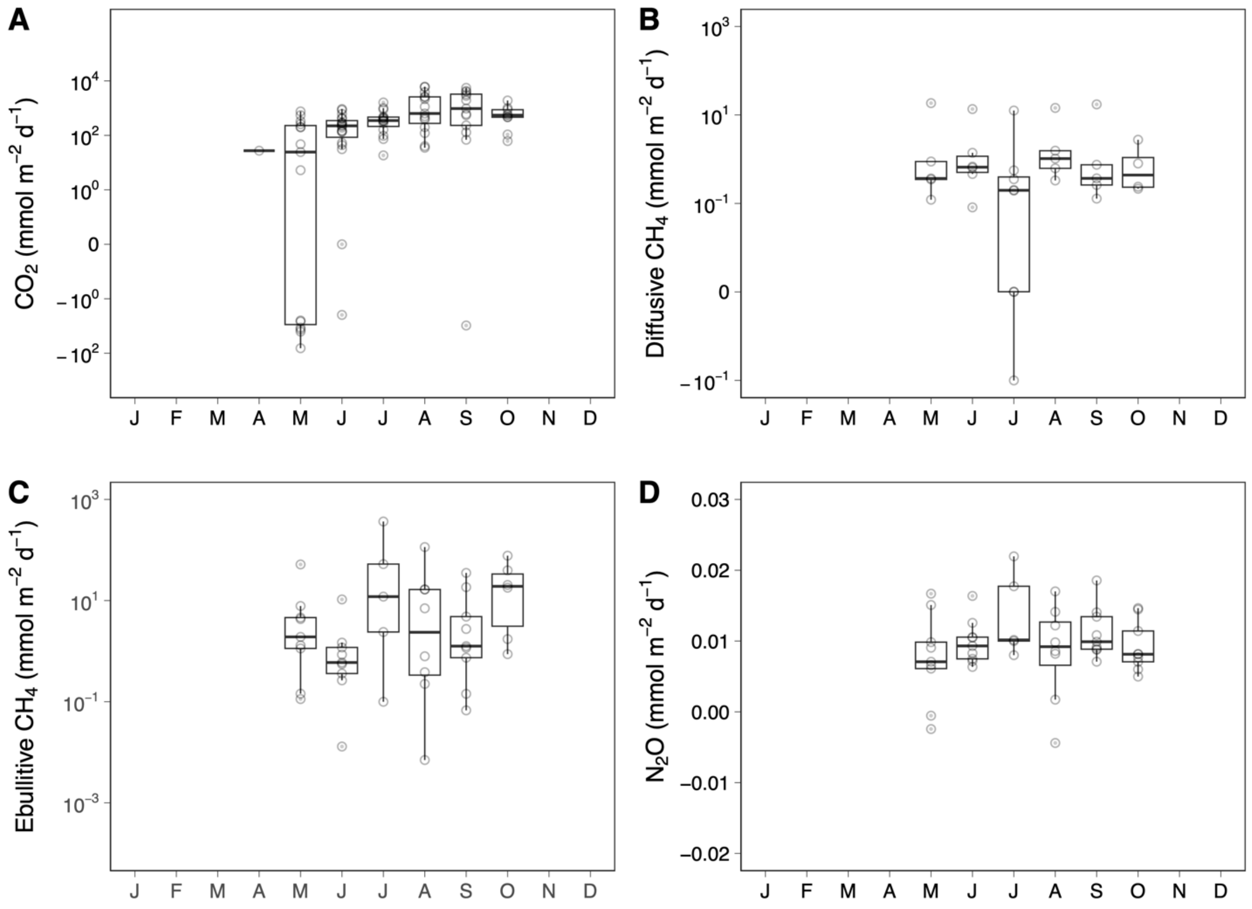

Fig. S8. **Seasonal variations of GHG flux rates in rivers using subsets of sites**. Sites with less than 5 months of observations were excluded in the analyses. Note that the y-axes for CO_2 and CH_4 fluxes are log-transformed.

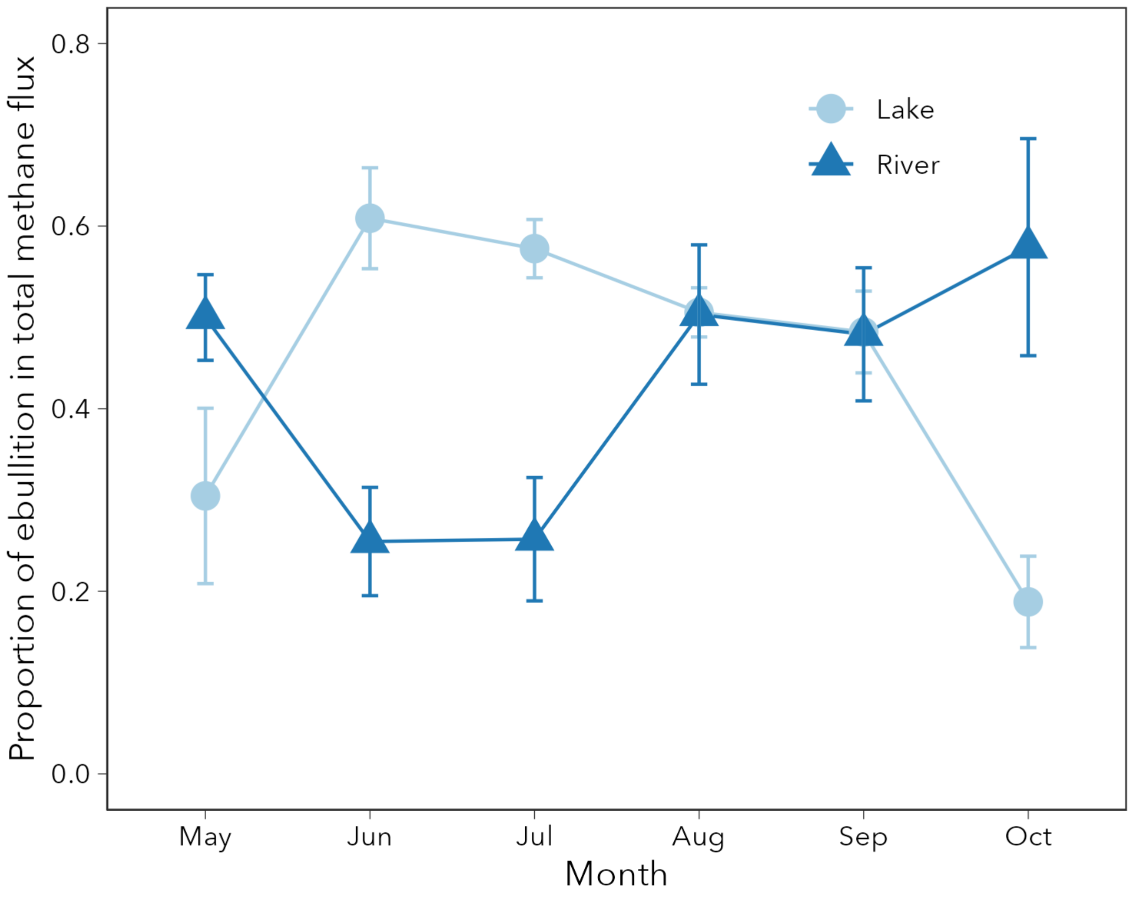

Fig. S9. The monthly average proportions of methane ebullitive flux to total flux. The portions were calculated using sites where both total and ebullitive flux are available.

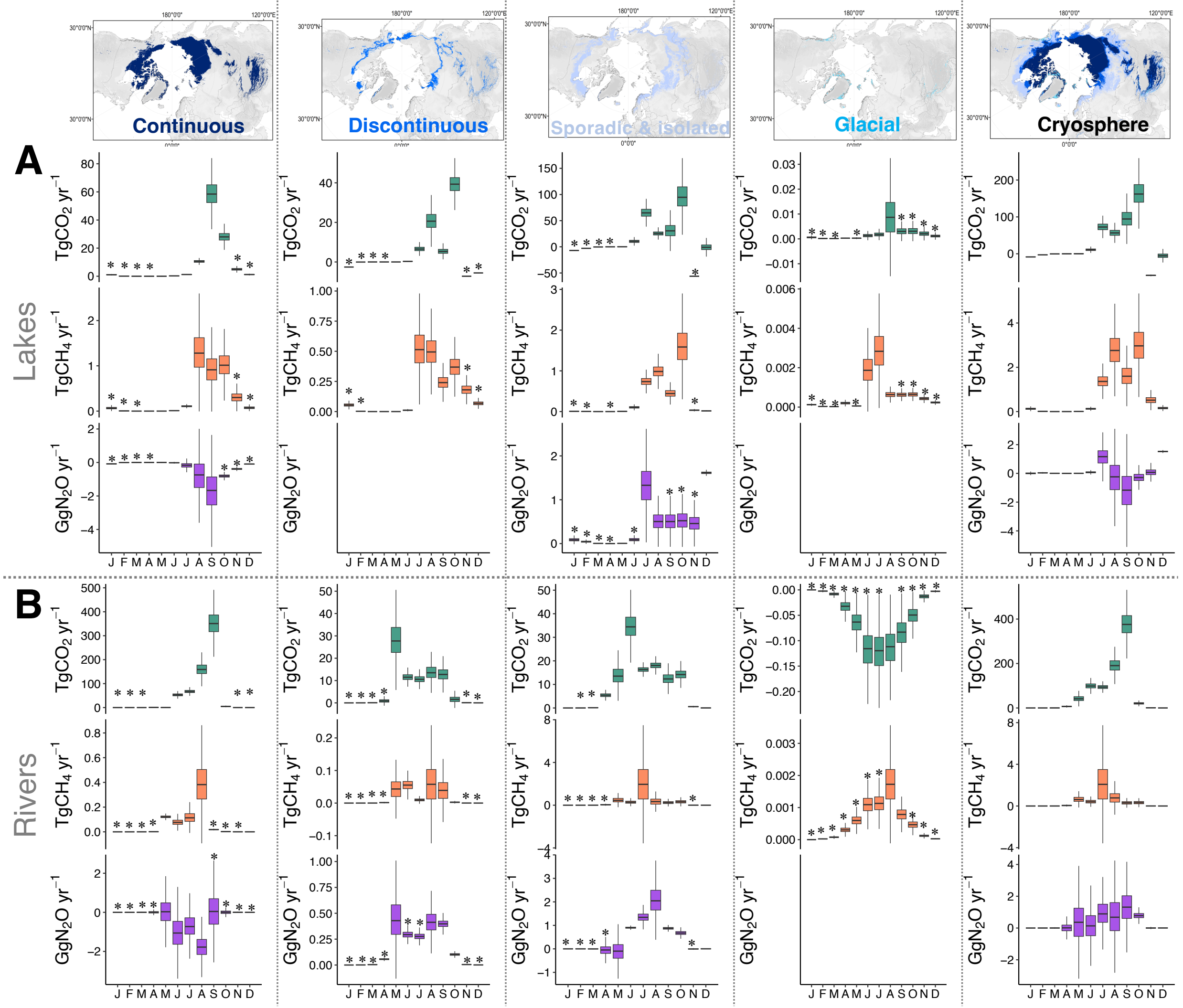

Fig. S10. Monthly GHG emissions of northern cryosphere inland waters segregated by cryosphere zonations. (A) Monthly GHG emissions in northern cryosphere lakes and reservoirs. **(B)** Monthly GHG emissions in northern cryosphere rivers and streams. Asterisks indicate that the emission of the month was upscaled with the annual lowest absolute values of the bootstrap statistics ($n < 3$, see Materials and Methods).

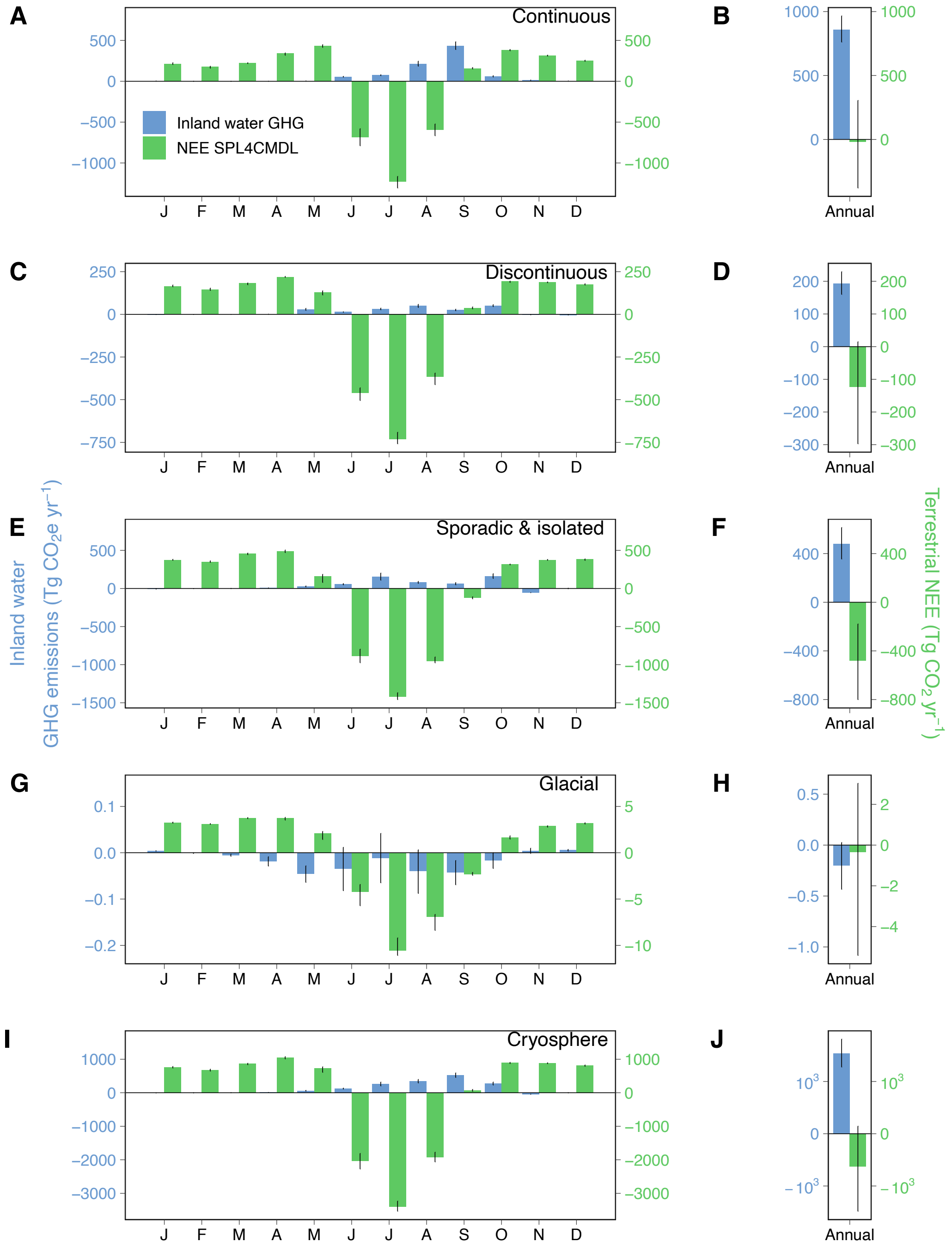

Fig. S11. Monthly (A-E) and annual (F-J) inland water CO_2e GHG emissions vs. land-atmospheric net ecosystem exchange of CO_2 (NEE) from the northern cryosphere. The inland water CO_2e GHG emissions are illustrated in GWP_{100}. The left Y-axes are GHG while the right Y-axes are NEE. For annual NEE (Tg CO_2 yr^{-1}) we use the SMAP L4 Global Daily 9 km EASE-Grid Carbon Net Ecosystem Exchange, Version 7 product (SPL4CMDL).

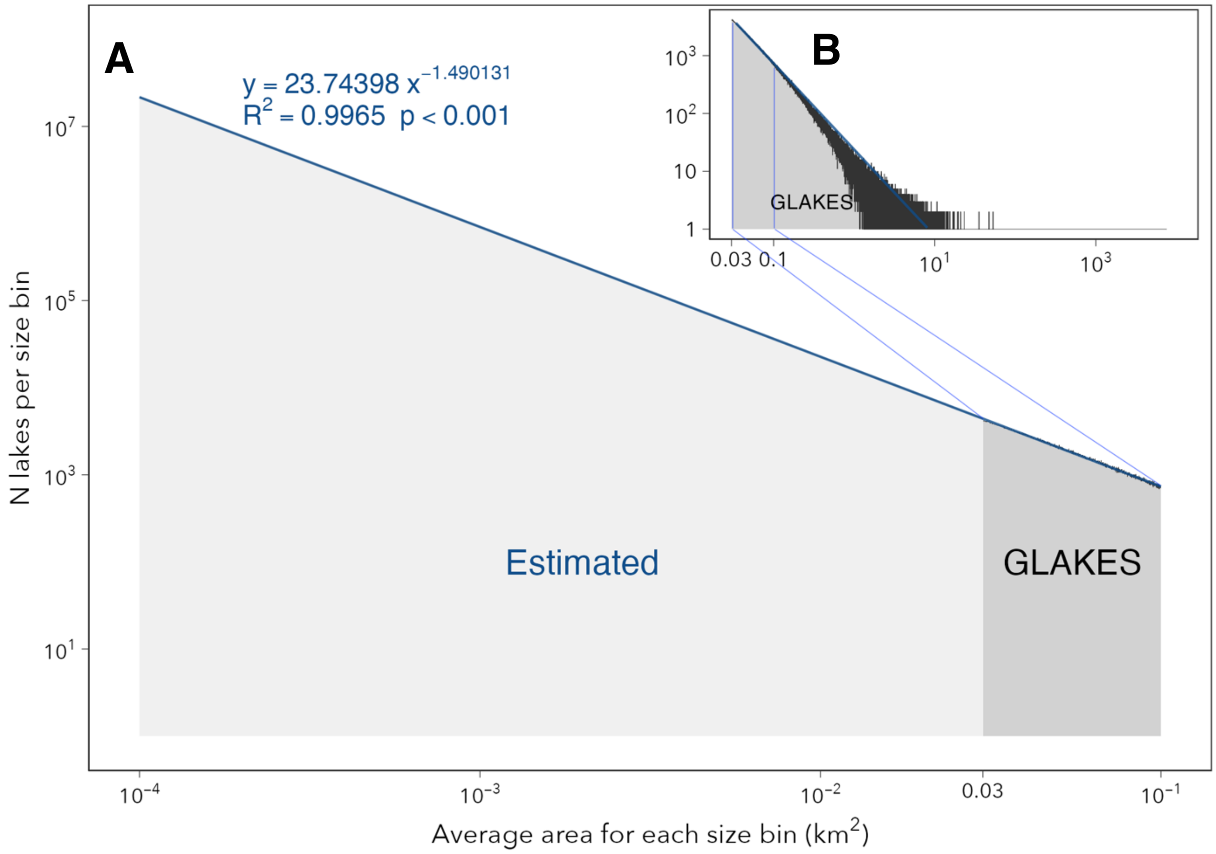

Fig. S12. Estimation of small lake area using log-log linear regression. The blue regression line is based on the 700 lake groups in the size range of 0.03 to 0.1 km^2. The average area of each size bin was calculated with the arithmetic mean of the lakes included in that bin. The estimated lake number of the 300 lake groups (54,797,691 lakes) in the size range of 0.0001 to 0.03 km^2 is based on the equation $y = 23.76341 \times x^{-1.490054}$. Each of the lake groups has a bin size of 0.0001 km^2. Panel (A) shows GLAKES of average bin area between 0.03 to 0.1 km^2 and estimated lake groups. Panel (B) shows all 53706 groups of GLAKES divided by a bin size of 0.0001 km^2. Note that the GLAKES data in this figure is limited to the northern cryosphere region.

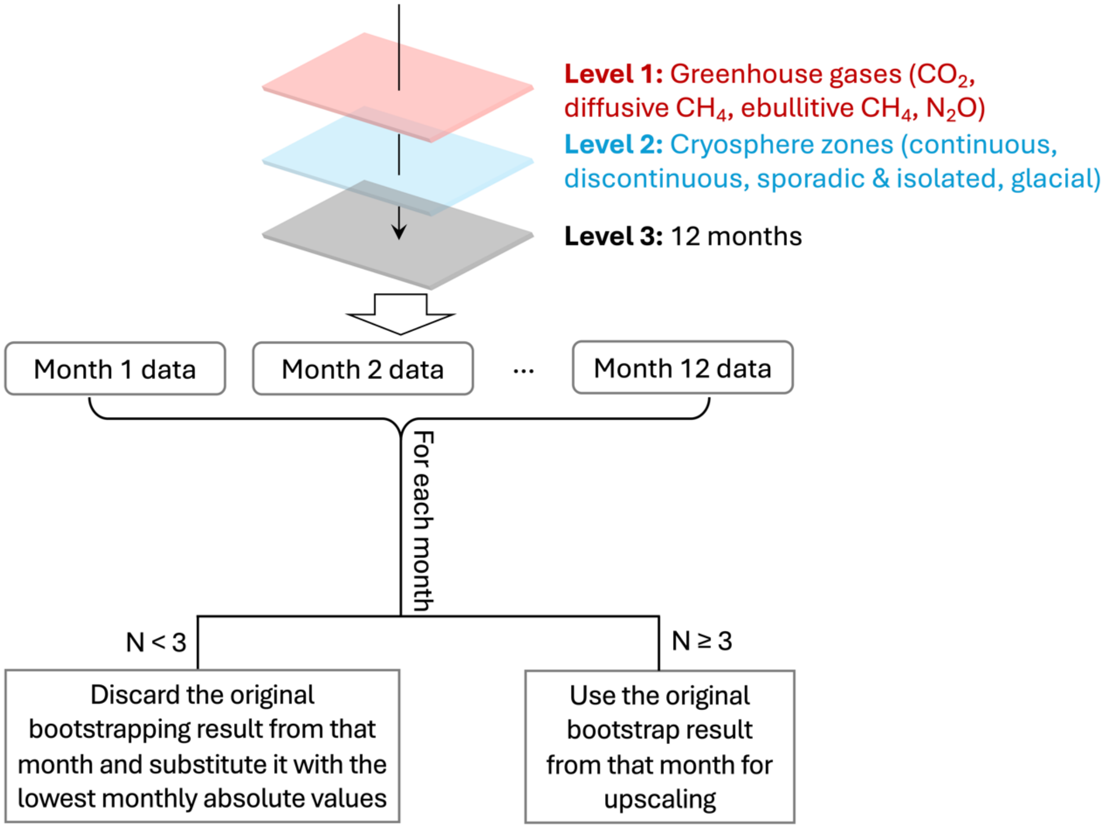

Fig. S13. Illustration of the multilevel bootstrapping approach.

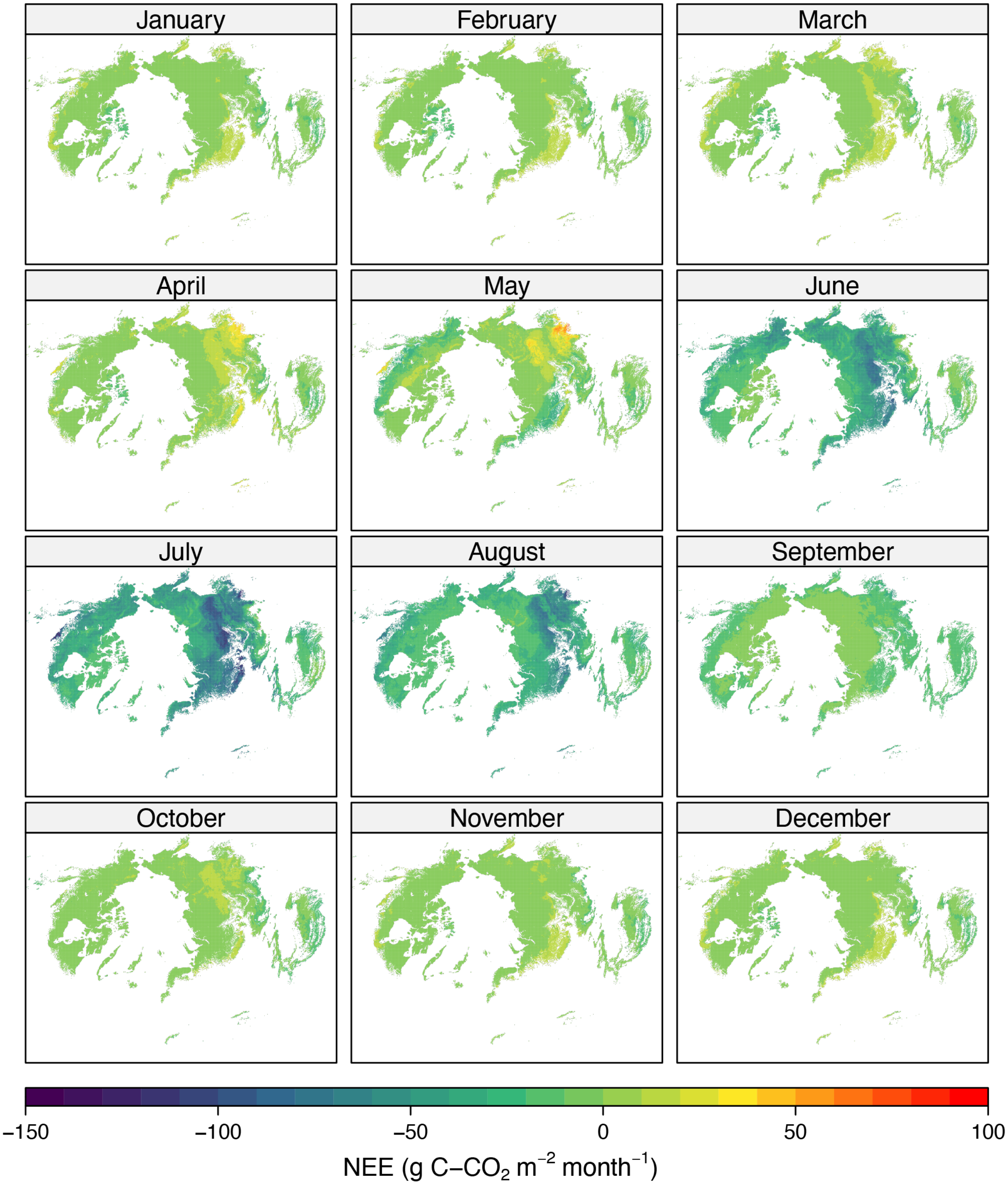

Fig. S14. Monthly mean net ecosystem exchange (NEE) rates across the northern cryosphere for the period April 2015 to March 2023. Data from SMAP L4 Global Daily 9 km EASE-Grid Carbon Net Ecosystem Exchange, Version 7 (https://nsidc.org/data/spl4cmdl/versions/7).

Table S1. Contemporary monthly open surface areas of northern cryosphere inland waters.

Water bodies	Cryosphere zonation	Jan	Feb	Mar	Apr	May	Jun	Jul	Aug	Sep	Oct	Nov	Dec
Lakes and reservoirs (km^2)	Continuous	66,063	3,894	91	73	116	3,020	41,301	285,487	539,705	531,766	260,008	61,534
	Discontinuous	32,661	1,605	24	21	35	2,981	46,774	119,543	137,661	137,968	102,779	37,532
	Sporadic & Isolated	78,466	40,742	3,739	159	942	78,599	329,425	434,670	449,279	451,009	408,498	191,809
	Glacial	304	67	47	41	106	317	660	961	1,198	1,187	810	432
	Cryosphere	177,494	46,308	3,901	293	1,200	84,917	418,160	840,660	1,127,842	1,121,930	772,095	291,307
Rivers and streams (km^2)	Continuous	0	195	762	4,478	53,876	92,428	84,415	84,303	79,768	7,354	1,005	172
	Discontinuous	0	83	342	5,502	23,781	28,744	26,155	26,484	25,825	9,648	435	84
	Sporadic & Isolated	0	118	1,249	36,109	72,468	74,455	69,899	69,811	67,781	48,757	2,250	245
	Glacial	0	71	217	897	1,703	3,213	3,218	3,006	2,310	1,330	352	72
	Cryosphere	0	467	2,571	46,986	151,828	198,840	183,687	183,603	175,684	67,090	4,041	573
Inland waters (km^2)	Continuous	66,063	4,089	854	4,551	53,992	95,447	125,717	369,790	619,473	539,120	261,013	61,705
	Discontinuous	32,661	1,688	366	5,522	23,816	31,725	72,929	146,027	163,485	147,617	103,214	37,616
	Sporadic & Isolated	78,466	40,860	4,987	36,269	73,410	153,054	399,325	504,480	517,060	499,766	410,748	192,055
	Glacial	304	138	264	937	1,809	3,530	3,877	3,967	3,508	2,517	1,161	505
	Cryosphere	177,494	46,775	6,471	47,279	153,028	283,757	601,848	1,024,264	1,303,526	1,189,020	776,136	291,880

Table S2. Statistics of measured GHG concentrations and fluxes. For each type of GHG in each zone, both both mean ± SD and median (Q1–Q3) are provided. Concentration unit: μmol L^{-1}, flux unit: mmol m^{-2} d^{-1}. NA, not available.

	GHGs	Statistics	Continuous	Discontinuous	Sporadic & Isolated	Glacial
Lake GHG concentrations (μmol L^{-1})	CO_2	mean ± SD	213.59±281.42	159.73±301.62	162.28±286.45	24.96±44.71
		median (Q1–Q3)	84.71 (29.71–304.28)	63.20 (31.60–144.78)	62.00 (37.01–154.00)	10.43 (3.07–23.14)
	CH_4	mean ± SD	26.26±124.66	24.39±131.34	9.14±44.27	1±2.72
		median (Q1–Q3)	0.76 (0.35–4.37)	1.50 (0.62–4.11)	0.74 (0.20–2.67)	0.19 (0.10–0.52)
	N_2O	mean ± SD	0.02±0.009	0.001±0.001	0.01±0.003	NA
		Median (Q1–Q3)	0.02 (0.01–0.03)	0.001 (0.0008–0.0015)	0.01 (0.009–0.112)	NA
Lake GHG fluxes (mmol m^{-2} d^{-1})	CO_2	mean ± SD	39.17±73.7	116.38±207.14	111.98±189.79	5.07±9.69
		Median (Q1–Q3)	18.12 (8.17–33.18)	38.63 (4.45–133.29)	51.58 (14.58–122.00)	2.44 (0.58–5.04)
	Diffusive CH_4	mean ± SD	5.85±10.31	7.54±14.56	5.98±10.18	2.06±4.32
		median (Q1–Q3)	1.22 (0.36–6.58)	1.59 (0.51–5.96)	1.67 (0.50–5.88)	0.22 (0.04–1.05)
	Ebullitive CH_4	mean ± SD	3.99±15.3	10.01±13.65	1.53±1.85	8.23±8.25
		median (Q1–Q3)	0.86 (0.12–3.61)	6.94 (1.39–14.02)	0.95 (0.29–1.99)	6.20 (0.52–14.15)
	N_2O	mean ± SD	0.001±0.005	NA	0.003±0.004	NA
		median (Q1–Q3)	0.002 (–0.002–0.005)	NA	0.002 (0.001–0.005)	NA
River GHG concentrations (μmol L^{-1})	CO_2	mean ± SD	106.46±114	151.16±161.17	84.69±101.18	33.36±22.31
		median (Q1–Q3)	72.00 (44.59–122.70)	108.25 (56.91–184.12)	41.58 (24.80–110.77)	29.98 (21.11–34.87)
	CH_4	mean ± SD	0.87±2.85	1.71±5.57	0.67±2.29	0.08±0.22
		median (Q1–Q3)	0.23 (0.09–0.77)	0.21 (0.17–0.99)	0.12 (0.02–0.47)	0.02 (0.01–0.07)
	N_2O	mean ± SD	0.02±0.009	0.001±0.001	0.01±0.003	NA
		median (Q1–Q3)	0.02 (0.01–0.02)	0.007 (0–0.013)	0.01 (0.009–0.013)	NA
River GHG fluxes (mmol m^{-2} d^{-1})	CO_2	mean ± SD	405.36±556.94	545.93±1150.87	243.34±430.14	27.89±28.26
		median (Q1–Q3)	147.74 (57.96–536.28)	240.62 (106.96–502.01)	109.72 (41.02–297.40)	-14.08 (-39.22–-12.50)
	Diffusive CH_4	mean ± SD	1.23±2.73	4.13±11.08	3.86±17.91	0.84±0.79
		median (Q1–Q3)	0.20 (0.02–0.99)	0.73 (0.05–3.27)	0.23 (0.04–1.55)	0.53 (0.26–1.26)
	Ebullitive CH_4	mean ± SD	2.39±4.91	1.99±6.08	9.66±21.84	NA
		median (Q1–Q3)	0.74 (0–1.47)	0.00 (0.00–0.00)	0.76 (0.35–3.61)	NA
	N_2O	mean ± SD	0.02±0.009	0.001±0.001	0.01±0.003	NA
		median (Q1–Q3)	0.006 (-0.01–0.01)	0.01 (0.009–0.016)	0.009 (0.007–0.014)	NA

Table S3. Seasonal CO_2e GHG emissions from northern cryosphere inland waters. Numbers are given as medians (Q1–Q3). NA, not available.

	Lakes		Rivers		Inland waters	
Month	Tg CO_2e yr^{-1} GWP20	Tg CO_2e yr^{-1} GWP100	Tg CO_2e yr^{-1} GWP20	Tg CO_2e yr^{-1} GWP100	Tg CO_2e yr^{-1} GWP20	Tg CO_2e yr^{-1} GWP100
January	1.52 (-0.72–3.87)	-5.11 (-5.96–-4.22)	NA	NA	1.52 (-0.72–3.87)	-5.11 (-5.96–-4.22)
February	-2.06 (-2.25–-1.89)	-2.59 (-2.66–-2.52)	0.04 (0.02–0.05)	0.03 (0.01–0.04)	-2.02 (-2.23–-1.84)	-2.56 (-2.65–-2.48)
March	-0.26 (-0.27–-0.25)	-0.28 (-0.28–-0.28)	0.3 (0.18–0.44)	0.2 (0.13–0.29)	0.04 (-0.09–0.19)	-0.08 (-0.15–0.01)
April	0.01 (0.01–0.01)	0 (0–0)	10.56 (7.44–13.82)	8.1 (6.2–10.08)	10.57 (7.45–13.83)	8.1 (6.2–10.08)
May	0.52 (0.42–0.61)	0.23 (0.19–0.27)	90.84 (68.48–117.55)	58.17 (45.13–73.66)	91.36 (68.9–118.16)	58.4 (45.32–73.93)
June	20.97 (17.15–25.08)	14.35 (11.67–17.19)	131.47(113.35–151.33)	109.85 (97.98–122.89)	152.44 (130.5–176.41)	124.2 (109.65–140.08)
July	181.33 (158.71–206.07)	109.65 (97.1–123.36)	259.49 (141.03–380.04)	150.12 (105.75–195.45)	440.82 (299.74–586.11)	259.77 (202.85–318.81)
August	276.48 (229.71–327.15)	131.05 (110.68–153.02)	252.47 (200.27–306.92)	211.58 (180.46–244.69)	528.95 (429.98–634.07)	342.63 (291.14–397.71)
September	221.23 (178.52–267.74)	137.11 (111.66–164.84)	400.19 (353.33–449.25)	384.24 (343.56–427.34)	621.42 (531.85–716.99)	521.35 (455.22–592.18)
October	398.52 (331.91–473.02)	242.07 (204.82–284.31)	46.33 (34.52–58.34)	29.39 (23.27–35.71)	444.85 (366.43–531.36)	271.46 (228.09–320.02)
November	-17.45 (-26.94–-7.51)	-44.61 (-48.22–-40.8)	0.88 (0.63–1.14)	0.71 (0.54–0.89)	-16.57 (-26.31–-6.37)	-43.9 (-47.68–-39.91)
December	7.66 (0.54–15.1)	-0.66 (-6.04–4.99)	0.08 (0.05–0.12)	0.05 (0.03–0.07)	7.74 (0.59–15.22)	-0.61 (-6.01–5.06)
**Annual**	**1088** (887–1309)	**581** (473–700)	**1193** (919–1479)	**952** (803–1111)	**2281** (1806–2788)	**1534** (1276–1811)

Table S4. Comparisons of terrestrial NEE and inland water GHG emissions from the northern cryosphere region. Numbers are given as medians (Q1–Q3).

Terrestrial NEE			Inland water GHG or carbon emissions		
NEE data source	NEE (Tg CO_2 yr^{-1})	NEE (Tg C yr^{-1})	CO_2e GWP_{20} (Tg CO_2e yr^{-1})	CO_2e GWP_{100} (Tg CO_2e yr^{-1})	C (CO_2-C+CH_4-C) emissions (Tg C yr^{-1})
SPL4CMDL	-623 (-1484–145)	-170 (-405–40)	2281 (1806–2788)	1534 (1276–1811)	324 (282–370)

Table S5. Seasonal GHG emissions (Tg CO_2e yr^{-1} at GWP20) from cryosphere lakes at different size classes. Numbers are given as medians (Q1–Q3). NA, not available.

Lake size classes	<0.1 km^2	0.1–1 km^2	1–10 km^2	10–100 km^2	100–1000 km^2	>1000 km^2	All lakes
January	2.38 (2.19 – 2.59)	0.79 (0.51 – 1.08)	0.02 (0.01 – 0.03)	0.08 (0.06 – 0.1)	0.78 (0.26 – 1.27)	10.06 (6.51 – 13.32)	14.11 (9.54 – 18.39)
February	0.003 (0.0028 – 0.0033)	0.001 (0.0006 – 0.0014)	0.0016 (0.0009 – 0.0023)	0.014 (0.011 – 0.017)	0.2 (0.07 – 0.32)	3.77 (2.44 – 4.99)	3.99 (2.52 – 5.34)
March	0.0019 (0.0016 – 0.0023)	0.0005 (0.0003 – 0.0007)	0.0008 (0.0005 – 0.0012)	0.0021 (0.0016 – 0.0026)	0.02 (0.01 – 0.03)	0.36 (0.24 – 0.48)	0.39 (0.25 – 0.52)
April	0.0043 (0.0035 – 0.0052)	0.0006 (0.0004 – 0.0008)	0.0007 (0.0004 – 0.001)	0.001 (0.0008 – 0.0013)	0.0063 (0.0021 – 0.0104)	NA	0.01 (0.01 – 0.02)
May	0.03 (0.02 – 0.03)	0.07 (0.07 – 0.08)	0.14 (0.09 – 0.2)	0.02 (0.02 – 0.03)	0.12 (0.08 – 0.17)	0.07 (0.05 – 0.09)	0.46 (0.33 – 0.6)
June	1.41 (1.25 – 1.59)	1.73 (1.42 – 2.08)	6.83 (3.94 – 9.75)	0.8 (0.63 – 0.97)	2.19 (1.27 – 3.06)	6.14 (4.45 – 7.75)	19.09 (12.96 – 25.19)
July	9.06 (8.43 – 9.73)	9.51 (8.61 – 10.46)	8.71 (5.54 – 11.59)	5.75 (5.38 – 6.14)	5.34 (1.89 – 8.63)	32.2 (23.83 – 40.21)	70.57 (53.68 – 86.75)
August	28.86 (26.88 – 30.85)	22.86 (19.79 – 26.09)	268.43 (150.28 – 388.26)	5.88 (4.2 – 7.57)	30.93 (21.73 – 39.56)	26.91 (17.88 – 35.35)	383.87 (240.76 – 527.69)
September	47.45 (42.25 – 52.9)	12.08 (9.41 – 14.94)	12.24 (8.67 – 16.14)	8.85 (7.26 – 10.62)	27.26 (18.03 – 35.91)	44 (32.08 – 55.38)	151.88 (117.7 – 185.89)
October	54.68 (48.46 – 61.61)	21.05 (15.82 – 26.54)	26.07 (19.37 – 33.01)	11.64 (9.28 – 14.07)	39.95 (25.84 – 54.15)	36.78 (26.12 – 46.95)	190.16 (144.88 – 236.33)
November	8.16 (7.5 – 8.86)	2.7 (1.74 – 3.69)	2.96 (1.72 – 4.32)	3.88 (2.98 – 4.78)	9.21 (3.04 – 15.07)	22.28 (14.43 – 29.52)	49.19 (31.41 – 66.24)
December	0.4 (0.37 – 0.44)	0.13 (0.09 – 0.18)	0.31 (0.18 – 0.45)	0.94 (0.73 – 1.16)	4.06 (1.34 – 6.64)	17.91 (11.6 – 23.72)	23.75 (14.3 – 32.6)
**Annual**	152.43 (137.35 – 168.61)	70.92 (57.45 – 85.13)	325.71 (189.79 – 463.75)	37.86 (30.55 – 45.46)	120.06 (73.56 – 164.83)	200.49 (139.63 – 257.78)	907.48 (628.33 – 1185.56)

Table S6. Seasonal GHG emissions (Tg CO_2e yr^{-1} at GWP100) from cryosphere lakes at different size classes. Numbers are given as medians (Q1–Q3). NA, not available.

Lake size classes	<0.1 km^2	0.1–1 km^2	1–10 km^2	10–100 km^2	100–1000 km^2	>1000 km^2	All lakes
January	0.88 (0.81 – 0.97)	0.38 (0.2 – 0.57)	0.01 (0.01 – 0.02)	0.07 (0.05 – 0.09)	0.26 (0.08 – 0.43)	6.47 (3.88 – 8.78)	8.08 (5.03 – 10.86)
February	0.0011 (0.001 – 0.0012)	0.0005 (0.0003 – 0.0007)	0.001 (0.0005 – 0.0016)	0.012 (0.009 – 0.015)	0.07 (0.02 – 0.11)	2.43 (1.46 – 3.29)	2.51 (1.49 – 3.42)
March	0.0007 (0.0006 – 0.0008)	0.0002 (0.0001 – 0.0004)	0.0006 (0.0003 – 0.0009)	0.0018 (0.0013 – 0.0022)	0.01 (0 – 0.01)	0.22 (0.14 – 0.3)	0.23 (0.14 – 0.32)
April	0.0015 (0.0012 – 0.0018)	0.0003 (0.0002 – 0.0004)	0.0004 (0.0002 – 0.0007)	0.0009 (0.0007 – 0.0011)	0.0021 (0.0007 – 0.0035)	NA	0.01 (0 – 0.01)
May	0.01 (0.01 – 0.01)	0.04 (0.03 – 0.04)	0.06 (0.04 – 0.08)	0.02 (0.01 – 0.02)	0.05 (0.04 – 0.07)	0.04 (0.03 – 0.06)	0.22 (0.16 – 0.29)
June	0.83 (0.73 – 0.94)	0.85 (0.7 – 1.02)	2.5 (1.49 – 3.52)	0.61 (0.49 – 0.73)	0.75 (0.43 – 1.05)	3.38 (2.22 – 4.49)	8.92 (6.07 – 11.75)
July	4.66 (4.31 – 5.03)	4.29 (3.81 – 4.78)	3.94 (2.81 – 4.97)	4.76 (4.48 – 5.04)	1.94 (0.72 – 3.12)	16.55 (11.15 – 21.7)	36.14 (27.29 – 44.65)
August	13.87 (12.84 – 14.91)	10.07 (8.58 – 11.61)	93.66 (53.44 – 134.43)	3.3 (2.27 – 4.25)	12.65 (9.24 – 15.87)	15.72 (9.67 – 21.18)	149.27 (96.05 – 202.25)
September	28.02 (25.18 – 30.98)	4.99 (3.42 – 6.64)	5.82 (3.52 – 8.33)	7.3 (6.1 – 8.54)	13.21 (9.69 – 16.52)	23.8 (15.72 – 31.49)	83.14 (63.64 – 102.49)
October	34.46 (30.65 – 38.72)	15.91 (12.71 – 19.23)	15.2 (11.68 – 18.88)	9.36 (7.57 – 11.13)	17.06 (11.76 – 22.41)	21.64 (13.86 – 29.05)	113.63 (88.23 – 139.42)
November	3.03 (2.76 – 3.3)	1.31 (0.69 – 1.94)	1.96 (0.92 – 3.09)	3.37 (2.51 – 4.24)	3.1 (0.96 – 5.13)	14.34 (8.6 – 19.46)	27.1 (16.45 – 37.17)
December	0.15 (0.14 – 0.16)	0.06 (0.03 – 0.1)	0.2 (0.09 – 0.32)	0.82 (0.61 – 1.03)	1.36 (0.42 – 2.26)	11.52 (6.91 – 15.64)	14.12 (8.21 – 19.51)
**Annual**	85.92 (77.43 – 95.03)	37.89 (30.18 – 45.94)	123.36 (74 – 173.64)	29.63 (24.12 – 35.09)	50.47 (33.37 – 66.99)	116.12 (73.65 – 155.43)	443.38 (312.75 – 572.12)
